# Effects of Darwinian Selection and Mutability on Rate of Broadly Neutralizing Antibody Evolution during HIV-1 Infection

**DOI:** 10.1371/journal.pcbi.1004940

**Published:** 2016-05-18

**Authors:** Zizhang Sheng, Chaim A. Schramm, Mark Connors, Lynn Morris, John R. Mascola, Peter D. Kwong, Lawrence Shapiro

**Affiliations:** 1 Department of Biochemistry and Molecular Biophysics and Department of Systems Biology, Columbia University, New York, New York, United States of America; 2 Laboratory of Immunoregulation, National Institute of Allergy and Infectious Diseases, National Institutes of Health, Bethesda, Maryland, United States of America; 3 Center for HIV and STIs, National Institute for Communicable Diseases of the National Health Laboratory Service, Johannesburg, South Africa; 4 Faculty of Health Sciences, University of the Witwatersrand, Johannesburg, South Africa; 5 Center for the AIDS Programme of Research in South Africa (CAPRISA), University of KwaZulu-Natal, Congella, South Africa; 6 Vaccine Research Center, National Institute of Allergy and Infectious Diseases, National Institutes of Health, Bethesda, Maryland, United States of America; University of Texas at Austin, UNITED STATES

## Abstract

Accumulation of somatic mutations in antibody variable regions is critical for antibody affinity maturation, with HIV-1 broadly neutralizing antibodies (bnAbs) generally requiring years to develop. We recently found that the rate at which mutations accumulate decreases over time, but the mechanism governing this slowing is unclear. In this study, we investigated whether natural selection and/or mutability of the antibody variable region contributed significantly to observed decrease in rate. We used longitudinally sampled sequences of immunoglobulin transcripts of single lineages from each of 3 donors, as determined by next generation sequencing. We estimated the evolutionary rates of the complementarity determining regions (CDRs), which are most significant for functional selection, and found they evolved about 1.5- to 2- fold faster than the framework regions. We also analyzed the presence of AID hotspots and coldspots at different points in lineage development and observed an average decrease in mutability of less than 10 percent over time. Altogether, the correlation between Darwinian selection strength and evolutionary rate trended toward significance, especially for CDRs, but cannot fully explain the observed changes in evolutionary rate. The mutability modulated by AID hotspots and coldspots changes correlated only weakly with evolutionary rates. The combined effects of Darwinian selection and mutability contribute substantially to, but do not fully explain, evolutionary rate change for HIV-1-targeting bnAb lineages.

## Author Summary

In an infected individual, the HIV-1 Env gene evolves at a rate of about 0.015 substitutions per site per year. Changes in viral epitopes in turn stimulate the co-evolution of recognizing antibody lineages. We previously showed that young antibody lineages can evolve at a rate ~10-fold faster than observed for HIV-1 and the rate of antibody evolution decreases over time. Here we investigate two factors, Darwinian selection and genetic mutability, which have been shown to influence evolutionary rates in other settings. We quantified both of these factors for three broadly HIV-1-neutralizing antibody lineages, and analyzed the association of these factors with changes in evolutionary rate. We found that Darwinian selection is a major factor in the slowing of evolutionary rate, while genetic mutability modulates antibody evolutionary rate weakly. Moreover, the combined effects of the two factors are unlikely to fully account for the slowing of antibody evolutionary rate.

## Introduction

Antibody affinity maturation is an iterative process of B cell proliferation, somatic hypermutation (SHM) of immunoglobulin variable gene regions, and selection. In germinal centers (GCs), B cells bearing antigen specific receptors (BCRs) take up antigens from the surface of follicular dendritic cell and present digested antigen peptides for recognition by CD4+ T follicular helper (Tfh) cells [1–5]. Once engaged with a Tfh cell, the B cell receives Tfh cell-derived cytokines and chemokines that are essential for survival and proliferation [6, 7]. Because there are limited numbers of Tfh cells in a germinal center, B cells compete for Tfh cell binding. B cells having BCRs with better binding affinity against a specific antigen will capture more antigen and thus present more antigen peptides on the cell surface, in turn leading to an enhanced chance to engage with Tfh cells [1, 2, 8]. Thus, beneficial mutations can be selected and accumulated in the immunoglobulin gene to promote BCR engagement [8, 9].

Broadly neutralizing antibodies (bnAbs) against HIV-1 have been shown to require high levels of SHM (up to 40%) for development of neutralization breadth and potency, and the maturation process usually takes several years [10–12]. This is because HIV-1 evolves quickly in the host, allowing the pathogen to escape antibody neutralization [10–13]. In response, cognate antibodies have to frequently rediversify their paratopes to keep engaged with the epitope. The necessary maturation cannot be accomplished in a single GC reaction, but rather requires cycles of reentry by memory B cells into new GCs for further proliferation and diversification [14, 15]. Over the long term, antibodies co-evolve with HIV-1 [10–12, 16], which can be approximated as a continuous process; however, the characterization of this evolutionary development is still limited.

The diversification of antibody V(D)J genes is initiated by activation-induced cytidine deaminase (AID)[3, 17]. AID mutates the antibody variable region at a rate of about 10^−3^ mutations per site per B cell generation [18]. However, only a portion of these mutations is non-deleterious and therefore have the potential to become fixed in the lineage. By approximating antibody evolution as a continuous process, the accumulation of these substitutions over time can be measured. This is termed the evolutionary rate. In our previous study, we estimated the evolutionary rates of three broadly neutralizing antibody (bnAb) lineages against HIV-1: CH103, VRC01, and CAP256-VRC26 (referred to hereafter as VRC26) [16]. During their respective study periods, the VRC26, CH103, and VRC01 lineages evolved with mean rates of approximately 7, 10 and 2 percent substitutions per nucleotide site per year respectively. We demonstrated the evolutionary rate of an antibody lineage to be at least comparable to that of the HIV-1 (~1.5 percent substitutions per site per year within host [16, 19]). However, the evolutionary rates of the VRC26 and CH103 lineages were found to be 3–5 fold faster than that of the VRC01 lineage, suggesting heterogeneity of evolutionary rates among lineages. Further analysis showed that the evolutionary rate of the VRC01 lineage was faster during the early part of the study period than during the later part. The observed inter- and intra- lineage evolutionary rate heterogeneity suggested that the rate of antibody evolution may be regulated by specific biological mechanisms.

Here, we estimate intra-lineage longitudinal evolutionary rate changes of VRC26 and CH103 lineages and compare these to the reported rate changes of the VRC01 lineage. The results confirm that a decreasing evolutionary rate is common to all three lineages. To help determine the mechanisms which modulate antibody lineage evolutionary rate, we examine selection pressure and variations in sequence mutability of these antibody lineages. We further discuss other possible factors that could underlie the slowing of evolutionary rates.

## Results

### Levels of somatic hypermutation under rapid antibody evolution

**Fig 1:**
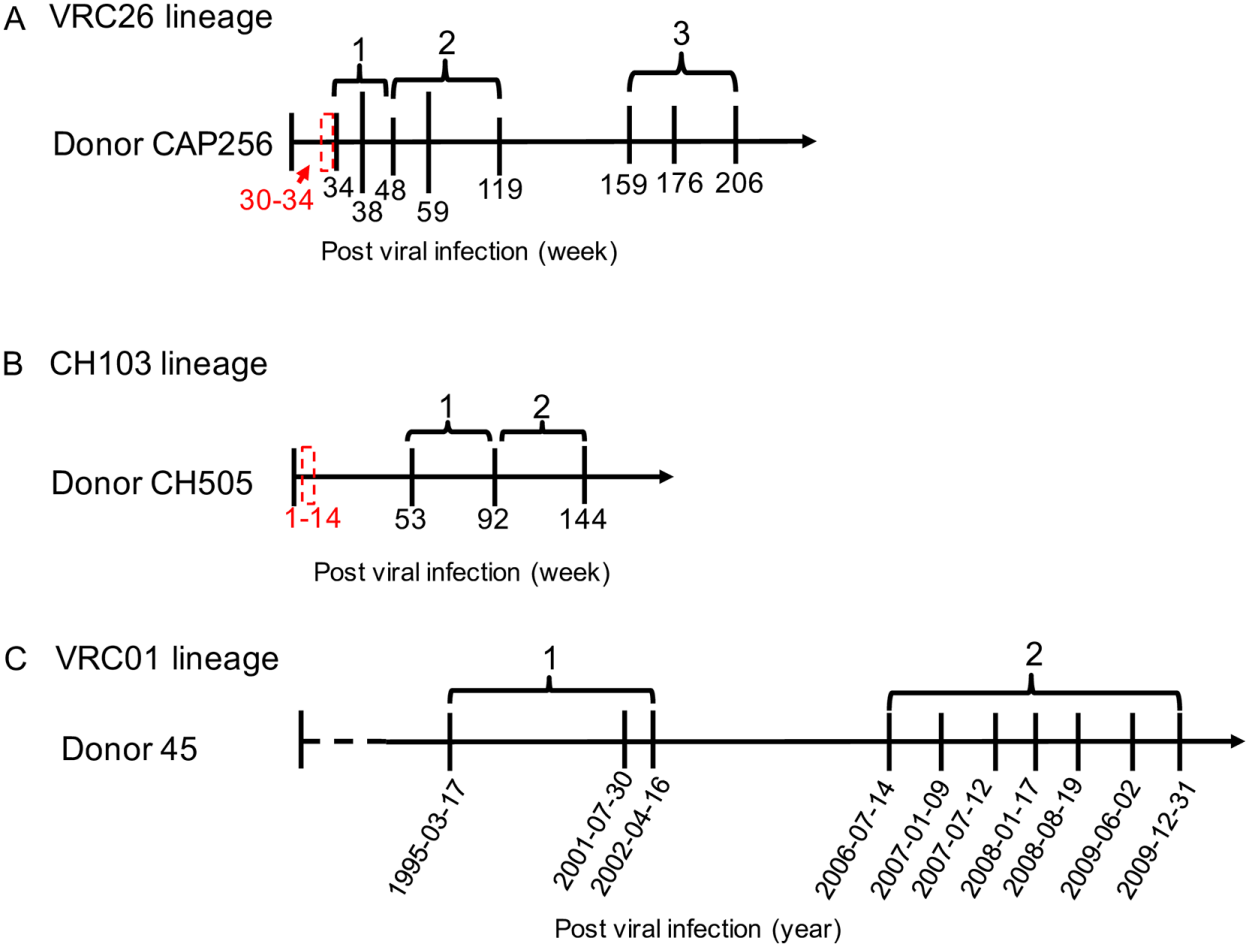
Study periods of the VRC26, CH103, and VRC01 lineages. To study the dynamics of evolutionary rate, selection pressure and mutability over time, we separated the time points of a lineage into two or three stages. (A) Time points of the CAP256-VRC26 lineage for which curated antibody variable region sequences were available. The eight time points were divided into three stages, which are listed above the time scale. (B) Time points of the CH103 lineage, with sequence data available at three time points, which were divided to two stages. (C). Study period of the VRC01 lineage. Since the exact date of HIV-1 infection is unavailable for this donor, the infection period before VRC01 lineage study initiation was represented with a dashed line. The approximate lineage initiation times for the VRC26 and CH103 lineages were labeled with red boxes.

A simple way to estimate how fast an antibody lineage evolves is to count the number of mutations accumulated in the variable region within a period of time. Usually, the level of SHM is measured as the divergence from a reference sequence such as the germline V gene. However, the drawback of using the germline V gene as a reference is that the complementary determining region 3 (CDR3) and the framework 4 region would be excluded from the calculation. In this study, we analyzed the changes of the levels of SHM over time for three broadly neutralizing antibody (bnAb) lineages (Fig 1) using published next-generation sequencing (NGS) data [10, 11, 16, 20]. We used the published unmutated common ancestors (UCAs) as references to calculate mean levels of mutation at each time point for the VRC26 and CH103 lineages [10, 11]. Because the evolution of the VRC01 lineage initiated several years before the study period (Fig 1), the UCA cannot be inferred from the available NGS data. Moreover, the lineage comprises several clades, which evolved independently for a long period of time after divergence from the UCA, with sequence divergence between clades reaching more than 30% [16]. We specifically examined the heavy and light chains of clades 03+06 and 08, the heavy chain of clade H3, and the light chain of clade L3 [16] and inferred the most recent common ancestor (MRCA) of each clade using MEGA6 [21]. These MRCAs were then used as references for calculating the levels of SHM.

**Fig 2:**
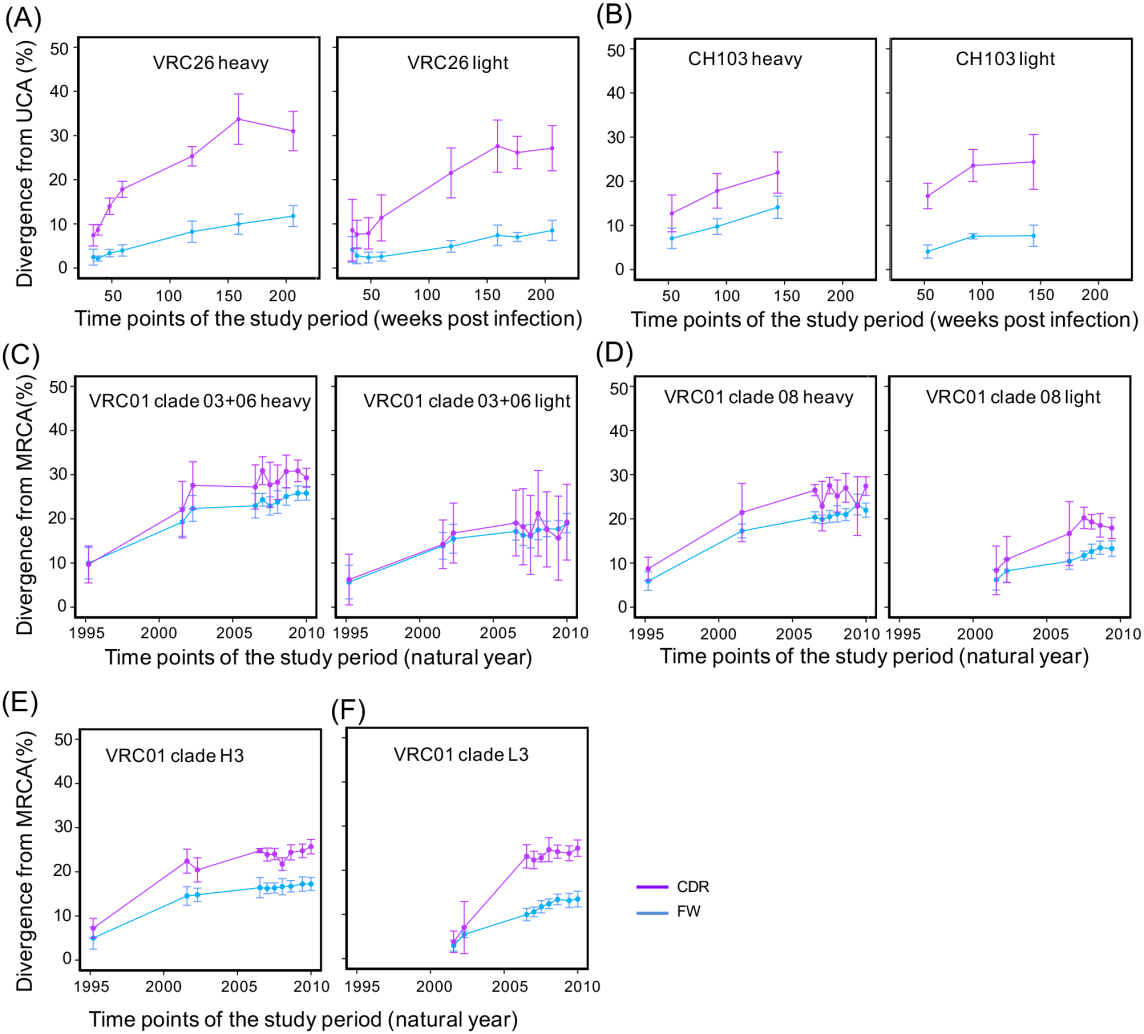
Measured somatic hypermutation levels from UCA or MRCA reach substitution saturation during antibody development. To demonstrate the dynamic changes of somatic hypermutation level (SHM) over time, the mean and 95% confidence interval of SHM was calculated for each time point of the three lineages. Stages from the same chain of a lineage were connected to reveal longitudinal trends. The plots of CDR regions were colored magenta while plots of FWRs were colored blue. (A) Longitudinal somatic hypermutation levels of VRC26 heavy (left) and light (right) chains. The measured SHM against UCA reached substitution saturation around 159 wpi. (B) Longitudinal somatic hypermutation levels of CH103 heavy (left) and light (right) chains. The SHM level from light chain UCA reached substitution saturation around 92 wpi, but the SHM level from heavy chain UCA appears to not reach substitution saturation. (C) Longitudinal somatic hypermutation levels of VRC01 clade 03+06 heavy (left) and light (right) chains. (D) Longitudinal somatic hypermutation levels of VRC01 clade 08 heavy (left) and light (right) chains. (E) Longitudinal somatic hypermutation levels of VRC01 clade H3. (F) Longitudinal somatic hypermutation levels of VRC01 clade L3. The measured SHM from the MRCAs of each clade reached substitution saturation around 2006.

The levels of mutations in complementary determining regions (CDRs) and framework regions (FWRs) of both heavy and light chain genes increased at early phases of evolution until substitution saturation, at which point later substitutions mostly occur at previously mutated sites. After substitution saturation, the levels of SHM are comparable between time points (Fig 2, roughly compared by the 95% confidence interval). Due to mutation bias and natural selection, only a portion of antibody positions can mutate easily, and the sequence divergence level from a reference plateaus once the most mutable positions have been changed. Thus, the divergence level does not accurately reflect the total number of accumulated mutations after saturation. Substitution saturation was reached at roughly 20–40% and 10–30% for CDRs and FWRs, respectively (Fig 2).

Longitudinally, the VRC26 lineage evolved for about 2.5 years (from about 30 weeks post infection (wpi) to 159 wpi) before reaching substitution saturation (about 34% and 10% SHM for CDRs and FWRs respectively) (Fig 2A). This evolutionary process also took about 2 years (from 14 wpi to 92 wpi) for CH103 light chain, while heavy chain did not reach saturation during the study period (Fig 2B). However, because we only have sequence data for three time points of the CH103 lineage, we have low confidence in the observed trends of substitution saturation. Furthermore, while it apparently took 7 years (from 1995 to 2002) for the VRC01 lineage to reach substitution saturation from the MRCAs of each clade (Fig 2C, 2D and 2E), this time period includes only 3 time points, again resulting in uncertainty. Thus there does not appear to be a fixed time frame for how long it takes antibodies to reach substitution saturation.

In summary, these analyses demonstrate that although antibody evolution leads to continuous accumulation of substitutions in the variable region, divergence from a reference sequence reaches a plateau within a few years. However, this may be the result of substitution saturation, rather than a decrease in the evolutionary rate. Because substitution saturation is affected by sequence length, nucleotide composition, mutation bias, and selection pressure [22, 23], it is difficult to accurately simulate the SHM process and calculate a theoretical limit. Genetic distance, which considers the above factors and infers multiple substitutions in a position via substitution models, may be more appropriate for characterizing the rate of antibody evolution.

### Examination of two methods for characterizing longitudinal changes of evolutionary rate

To examine longitudinal intra-lineage evolutionary rate heterogeneity, we estimated evolutionary rate changes for our samples using the program BEAST2 [24] with relaxed log-normal molecular clock model. Through Markov Chain Monte Carlo (MCMC) simulation, BEAST2 first enumerates many trees via topological reshuffle of an initial tree, and searches for a set of trees with high posterior probabilities to explain the relations of a temporal sequence alignment sample. Then the program calculates a mean rate of evolution for each tree assuming a preassigned molecular clock model and population coalescent model, and finally estimates the mean evolutionary rate and the 95% highest posterior density (HPD) interval of the mean rate for the dataset. By using a set of trees with high posterior probabilities, BEAST2 reduces the effect of phylogenetic uncertainty on evolutionary rate estimation.

In this study, we evaluated the performance of two methods for characterizing evolutionary rate changes over time. In the time-bin method, we separated the time scaled Bayesian maximum clade credibility (MCC) trees (from BEAST2 simulation) along the time axis into bins, and estimated the evolutionary rate for each bin (mean evolutionary rate only, no 95% HPD) from all tree branches within the bin [25]. (A time scaled Bayesian MCC tree is one in which the length of a tree branch corresponds to the estimated elapsed time between nodes, rather than to genetic distance.) Alternatively, for the stage method, the sequences were divided into stages based on the dates of collection (see Materials and Methods). In this case, the mean evolutionary rate of each stage was estimated using BEAST2 simulation separately, and the changes of the mean evolutionary rates between stages were used to reflect the rate changes of the dataset.

To compare the two methods, we simulated the evolution of a gene under a constant evolutionary rate (CR dataset) and selected sequences from eight time points. We also duplicated this data and changed the time labels of the sequences to generate a dataset with decreasing evolutionary rate (DR dataset, see Materials and Methods). Because the estimation of evolutionary rate is sensitive to substitution saturation, we examined substitution saturation signal in the two datasets and showed that the datasets are not substitution saturated (S1 Table). We then examined how accurately the two methods can estimate the changes in the evolutionary rate of the DR dataset over time.

**Fig 3:**
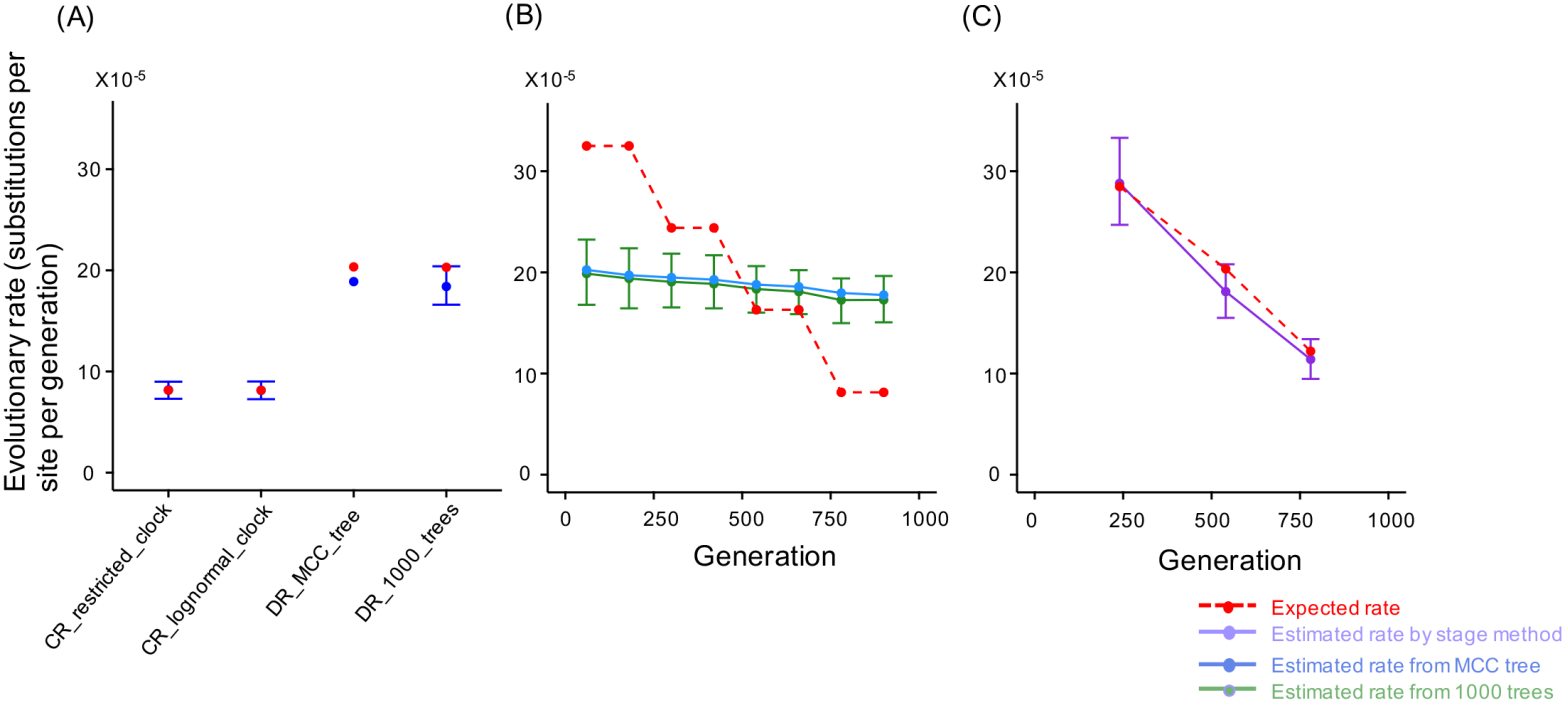
The stage method is better than the time-bin method for estimating evolutionary rate dynamics. (A) The evolutionary rate for the constant-rate (CR) dataset was estimated using both restricted and relaxed log-normal clock models (labeled CR_restricted_clock and CR_lognormal_clock respectively). The estimated mean evolutionary rates from the two models are in good agreement. The mean evolutionary rates of the decreasing rate (DR) dataset estimated from the MCC tree and the 1000 time scaled Bayesian trees (labeled DR_MCC_tree and DR_1000_tree respectively) are highly consistent with the expected rates (red dots) derived from the calculated evolutionary rate of the CR dataset (see Materials and Methods). (B) The mean evolutionary rates for the eight time bins of the DR dataset were estimated from a single MCC tree (blue) and the mean and the 95% highest probability density (HPD) intervals estimated from 1000 time scaled Bayesian trees (green). The estimated evolutionary rates for each bin are significantly different from the expected rate (red). (C) The expected rates (red) of the three stages of the DR dataset are within the estimated 95% HPD of the mean evolutionary rates (purple), suggesting the stage method is reliable for characterizing evolutionary rate changes over time.

To evaluate the performance of the time-bin method, we first estimated the evolutionary rate for the CR dataset using both the restricted molecular clock and the relaxed log-normal clock models. The estimated evolutionary rates from the two models are highly consistent (Fig 3A, left and S3 Fig). A similar evaluation of the DR dataset using the relaxed log-normal clock model matched the simulated rates both for the point estimate from the MCC tree and the 95% HPD range (Fig 3A, right).

When the DR dataset was evaluated with the time-bin method, the results underestimated the evolutionary rates for the early bins and overestimated the rates for the late bins (Fig 3B). The 95% HPD intervals were consistent with the point estimates from the MCC tree, and the true simulated rates were not within the estimated 95% HPD interval for any bin (Fig 3B). For the stage method, however, the expected rates are within the estimated 95% HPD intervals for all three stages (Fig 3C). This suggests that the relaxed log-normal clock model can accurately estimate the mean evolutionary rate of a dataset, but may not be able to estimate the evolutionary rate accurately for each local branch of the tree. This is possibly because the dramatic variations in evolutionary rate between tree branches may violate the mathematical assumptions of the model. In addition, the random local clock model may be a better choice for evolutionary rate estimation when rate variations exist between branches [25]. However, the simulations with random local clock model failed to converge and the estimated log posterior probability increased to positive values, which should be impossible (S1 Fig). Although we are unsure why BEAST produces meaningless numbers in this case, it is probably because the model is only valid for datasets with certain underlying tree structures [25]. In summary, the stage method is a better choice for characterizing evolutionary rate changes, and it is used for the evolutionary rate estimation in this study.

### Evolutionary rate decrease of broadly neutralizing antibody lineages

**Fig 4:**
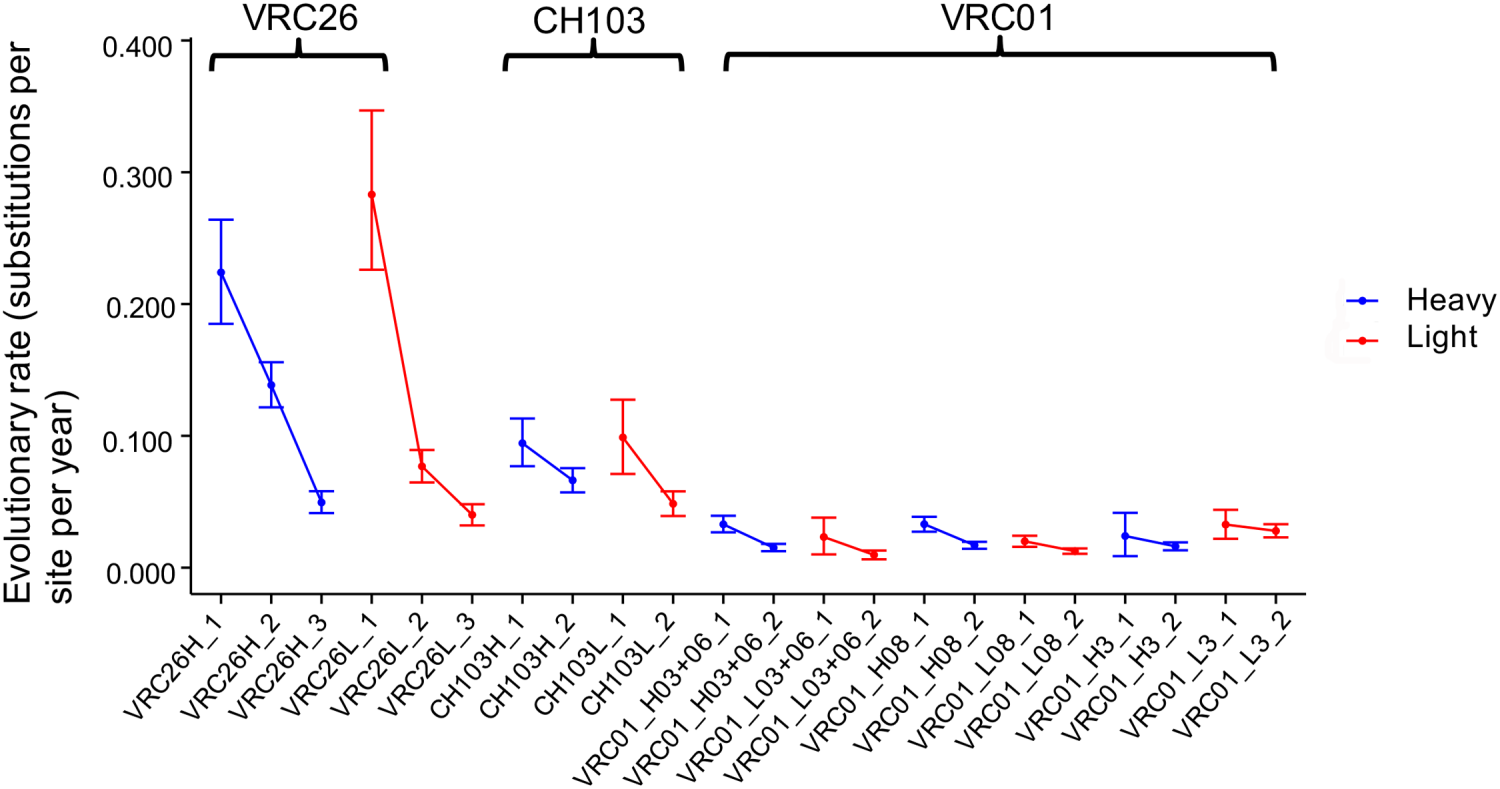
The evolutionary rates of the three antibody lineages decrease over time. The evolutionary rates for the stages of the three lineages were calculated using BEAST2. The mean evolutionary rates for the VRC26, CH103, and VRC01 lineages decreased about 80%, 45%, and 35% respectively over their study periods. However, the observed evolutionary rate decrease is not statistically significant for clade 08 light chain, clade H3, and L3. Each stage is labeled with the format of lineage name, chain name, and stage. Because clades of the VRC01 lineage showed more than 30% divergence from each other, we chose four representative clades (03+06, 08, H3, and L3) and calculated their evolutionary rates separately. The mean and 95% HPD are shown for each stage. Stages from the same chain of a lineage are connected to reveal longitudinal trends. The evolutionary rates of heavy chains and light chains are colored blue and red respectively.

Because two or more time points are required to have an accurate estimation of evolutionary rate, we separated the study periods of VRC26 (eight time points) and CH103 lineages (three time points) into 3 and 2 arbitrary stages, respectively (Fig 1), and estimated the evolutionary rate for heavy and light chains of each stage. The sequence data of 92 wpi of the CH103 lineage, which is at the boundary of stage 1 and stage 2, were used to estimate evolutionary rates of both stages. Similarly, the sequence data from 48 wpi of the VRC26 lineage were used to estimate evolutionary rates for both stage 1 and 2. Antibody sequences from 34 wpi, 159 wpi, and 193 wpi [20, 26], which were not available for our previous study [16], were added to the analysis of the VRC26 lineage. We also selected four clades of the VRC01 lineage, two of which (03+06 and 08) have both heavy chain and light chain data, one of which (H3) has only heavy chain data, and one of which (L3) has only light chain data. We used these clades to re-calculate the evolutionary rates for the two stages we reported previously [16]. Consistent with our previous study on the VRC01 lineage, both heavy chain and light chain of the VRC26 and CH103 lineages show significant evolutionary rate decrease over time (Fig 4).

Because evolutionary rate estimation is sensitive to substitution saturation, we performed substitution saturation test for datasets used in this study using DAMBE5 [22, 23], which showed that substitutions are significantly far from saturation in any of our datasets (P<0.01, S1 Table). Thus, the estimated evolutionary rates are not severely affected. This is mainly due to three reasons. First, the sequence divergence between groups of sequences from consecutive time points is far below the level of substitution saturation. Second, the separation of time points into stages reduced the effect of substitution saturation, because the MRCA of each stage is more recent than the overall MRCA. Third, the substitution model used for evolutionary rate calculation incorporates the possibility of multiple substitutions at a position.

The study period of the VRC26 lineage is from the 34th wpi to the 206th wpi. The available data showed that the lineage was initiated between 30 wpi and 34 wpi (Fig 1A) [10]. Thus the three stages correspond approximately to the 4th to 18th weeks, 18th to 89th weeks, and 129th to 176th weeks of antibody development, respectively. The first stage of the VRC26 lineage heavy chain showed a mean evolutionary rate as high as 0.224 substitutions per site per year (Fig 4). This rate is about 15-fold higher than the reported intra-donor evolutionary rate for the *Env* gene [16, 19]. This suggests that B cells are capable of dramatic diversification of antibody variable regions within the first few weeks post lineage initiation. However, the second and third stages showed a mean rate of 0.139 and 0.049 substitutions per site per year, which are 62% and 22% of the rate of the first stage, respectively. The 95% HPD of the evolutionary rates of the three stages showed no overlap, indicating statistically significant differences. Consistent with heavy chain, the evolutionary rate of light chain decreased 86%, from 0.283 to 0.040 substitutions per site per year. Nonetheless, the evolutionary rates of the third stages of both heavy and light chains are still significantly faster than the reported rates for the HIV-1 *env* gene [16, 19].

Similar to the VRC26 lineage, the 95% HPD of the first stage of both heavy and light chains of the CH103 lineage do not overlap with the 95% HPD of their respective second stage (Fig 4), suggesting the lineage evolved significantly faster during the first stage than the second. CH103 lineage members were isolated in CHAVI donor 505 as early as 14 wpi [11], although, due to the limited number of sequences available at 14 wpi, we could not determine the evolutionary rate for this earliest stage of the lineage development. By assuming the development of the CH103 lineage was initiated between 1 and 14 wpi, the first stage of our study period (53 to 92 wpi) is roughly from the 39th to 92nd weeks of antibody development. The second stage (92 to 144 wpi) is roughly from the 79th to 144th weeks of antibody development. The two stages of the CH103 lineage correspond approximately to the second and third stages of the VRC26 lineage. Interestingly, the evolutionary rates of the two stages of CH103 light chain are comparable to that of the second and third stages of VRC26 light chain, but heavy chain showed significant differences between the corresponding stages of the two lineages (Fig 4, middle).

Consistent with the VRC26 and CH103 lineages, both heavy and light chains from the VRC01 lineage showed significant decreases in evolutionary rate over time except clades L08, H3, and L3 (Fig 4, right). For all clades, the second stage showed an evolutionary rate about 50% of that of the first stage. However, the evolutionary rates of the first stage of VRC01 clades are themselves only about 50% of the evolutionary rates of the last stages of the other two lineages. This is likely because the VRC01 lineage evolved for an unknown number of years before the beginning of the study period [16] and the evolutionary rate therefore had a longer time to decay than for the other two lineages. Interestingly, although the VRC01 lineage clades had diverged from each other at least ten years before the end of the study, their evolutionary rates remain comparable, implying similar mechanisms of evolutionary regulation [16].

### Evolutionary rates of CDR and framework regions

**Fig 5:**
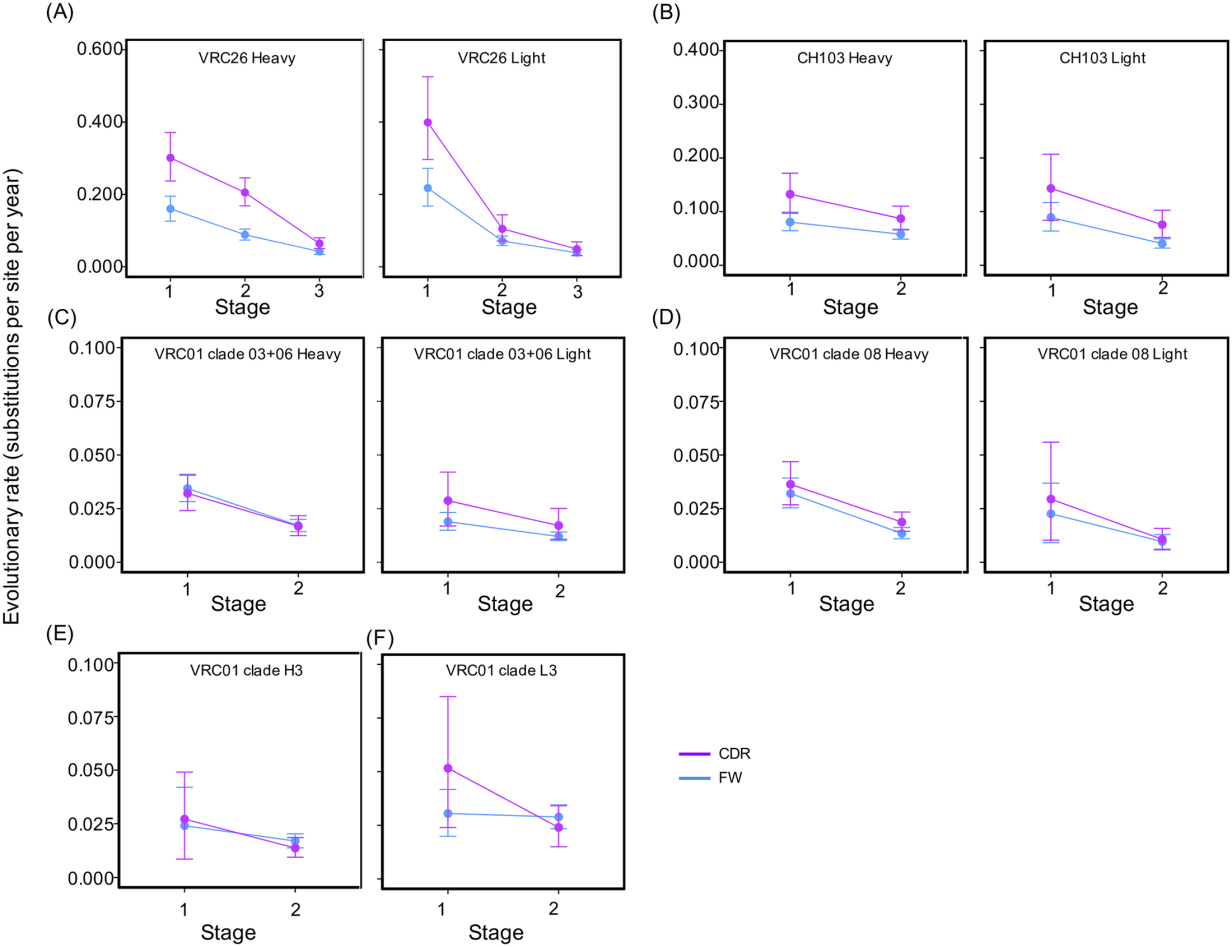
The evolutionary rates of CDRs and FWRs of the three lineages decrease over time. The mean and 95% highest probability density interval (HPD) are shown for each stage. Stages from the same chain of a lineage are connected to reveal longitudinal trend. The plots of CDR regions are colored magenta while plots of FWRs are colored blue. (A) The mean evolutionary rates for the CDRs and FWRs of the VRC26 heavy (left) and light (right) chains showed about 80% decrease over the study period. (B) The mean evolutionary rate dynamics for the CDRs and FWRs of the CH103 heavy (left) and light (right) chains showed about 45% decrease over the study period. (C) Evolutionary rate dynamics for the CDRs and FWRs of the VRC01 clade 03+06 heavy (left) and light (right) chains showed about 45% decrease over the study period. But the evolutionary rate decrease for light chain CDRs is not significant. (D) Evolutionary rate dynamics for the CDRs and FWRs of the clade 08 heavy (left) and light (right) chains. The decreases in evolutionary rates are only significant for both regions in heavy chain. (E) and (F) show evolutionary rate dynamics for the CDRs and FWRs of the VRC01 clade H3 and L3, respectively. No significant decrease in evolutionary rate was detected for the two clades.

Since CDRs and FWRs play different functional roles and FWRs undergo stronger negative selection pressure [27, 28], we estimated the evolutionary rates separately for CDRs and FWRs to examine whether the evolutionary rate dynamics are different between them. The results showed that the evolution of both CDRs and FWRs slowed over time for the VRC26 and CH103 lineages (Fig 5). For example, the CDRs of VRC26 heavy chains showed about 6-fold of rate decrease from the first stage to the third stage. The FWRs also showed about 4-fold of evolutionary rate decrease. For the VRC01 lineage, the heavy chains of clades 03+06 and 08 and the light chain FWRs of clade 03+06 showed significant evolutionary rate decreases. The consistent decrease of evolutionary rate in both CDRs and FWRs strengthens our hypothesis that evolution of the entire variable region is systematically regulated [16]. We also observe that CDRs evolve about 1.5- to 2-fold faster than FWRs at all stages of development for the VRC26 and CH103 lineages. This expected result is due to stronger functional selection pressure and lower mutability in the FWRs, as demonstrated below. Surprisingly, however, the evolutionary rates in the CDRs of the VRC01 lineage are similar to those in the FWRs (Fig 5C–5E), which may be due to strong negative selection pressure in CDRs (see Selection pressure section below). In addition, the sequence of CDRs is shorter than that of FWRs, making it possible for CDRs to achieve higher evolutionary rate with a smaller number of substitutions.

### Distinguishing alternative mechanisms for evolutionary rate decrease

The consistent decrease in the evolutionary rate across all three antibody lineages suggests there are mechanisms regulating antibody evolution systematically. Three mechanisms that may account for the substitution rate change are listed below.

The first hypothesis is what we term the affinity maturation selection (AMS) model [29, 30]. As antigen-binding affinity becomes high, fewer amino acid-changing substitutions are likely to increase affinity. Instead of positive selection, negative selection becomes dominant and removes most non-synonymous mutations. Below, we show that this process plays a significant role in the decrease of the evolutionary rate.

A second possible mechanism is that the rate of mutations generated by AID decreases over time. This hypothesis is based on the observation that AID selectively mutates hotspot nucleotide motifs and avoids mutating coldspot motifs [31, 32]. It is possible that the hotspot motifs are consumed during the fast evolution of the early phase such that AID is less capable of mutating antibody genes at later phases. Below, we show that the correlation between mutability and evolutionary rate is weak.

A third possible mechanism is that the frequency of B cell proliferation slows over time due to many possible factors such as viral escape and decrease of viral epitope abundance. For example, as escape mutations fix in the viral population, B cells producing receptors targeting the old epitope can no longer take up antigen efficiently, which reduces the chance of engagement with Tfh cells. Thus, B cells receive less cytokines and chemokines that are required for proliferation. The proliferation of B cell slows and therefore fewer substitutions accumulate in the later phases of antibody evolution. While the third mechanism cannot be examined by studying antibody sequences, we address it further in the discussion section.

### Functional selection pressure on CDRs and Framework regions

To examine the contribution of the AMS model to evolutionary rate decay, we first estimated selection strength changes for each time point of the three lineages using the program BASELINe [28]. BASELINe takes into account mutation targeting bias and substitution bias when calculating antibody selection strength. BASELINe further normalizes the measured selection strength so that the selection strength between time points and between lineages can be compared. Because selection strength is estimated from the log odds ratio of non-synonymous mutations/synonymous mutations, a proper reference sequence is important for counting each type of substitutions. In particular, the estimated selection strength of a sequence at a time point should contain little selection signal of previous time points. To approach this, we first built a phylogenetic tree using available sequences of all time points for each chain of a lineage. Then we inferred the sequences for all internal nodes using MEGA6 [21]. Starting from a selected terminal branch, we searched for the first internal node containing a terminal branch from an earlier time point. The sequence of the internal node is used as reference to remove substitutions evolved at/before earlier time points. To quantify the correlation between selection pressure and evolutionary rate, we also estimated the selection strength for each stage of each lineage and calculated intra-lineage correlations between evolutionary rate and selection strength.

**Fig 6:**
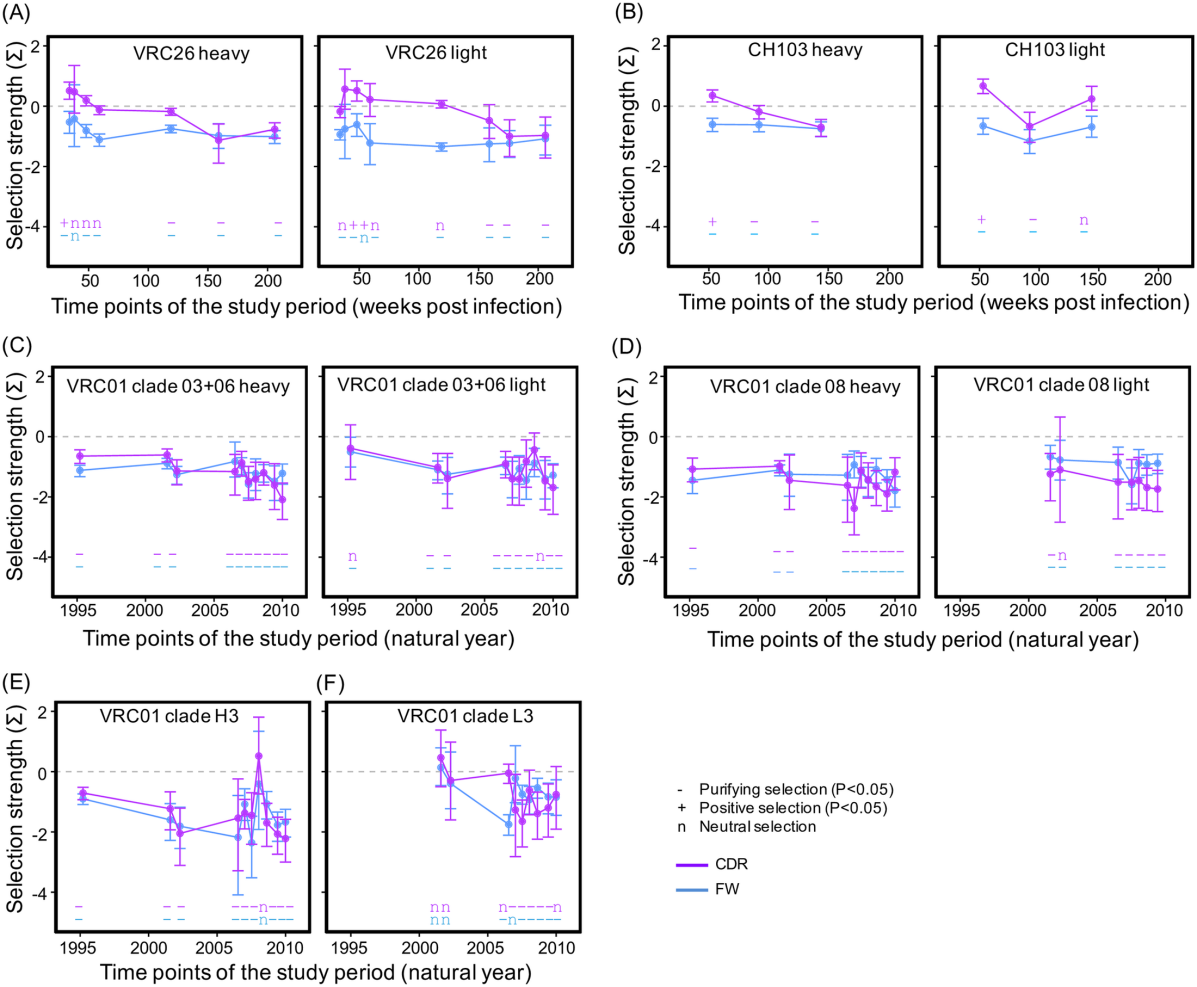
Selection pressure dynamics for all time points of the three lineages. (A) Selection pressure changes of the VRC26 lineage heavy (left) and light (right) chains. (B) Selection pressure changes of the CH103 lineage heavy (left) and light (right) chains. (C) Selection pressure changes of the VRC01 clade 03+06 heavy (left) and light (right) chains. (D) Selection pressure changes of the VRC01 clade 08 heavy (left) and light (right) chains. (E) Selection pressure changes of the VRC01 clade H3. (F) Selection pressure changes of the VRC01 clade L3. The selection strength for each time point of a lineage chain was measured using BASELINe. The same datasets used for evolutionary rate calculation were used to calculate selection strength. The mean and 95% HPD interval of selection strength for the CDRs (magenta) and FWRs (blue) were calculated separately. The statistical significance of the measured selection strength is shown on the bottom of the plot with ‘-’, ‘+’ and ‘n’ denoting negative selection, positive selection, and neutral selection, respectively.

The results showed that selection strength dynamics on CDR regions are consistent with reported co-evolution between antibody and virus. The heavy chain CDRs of the VRC26 lineage showed significant positive selection at 34 wpi, neutral selection from 38 wpi to 59 wpi, and significant negative selection from 119 wpi to 206 wpi (Fig 6A). The relaxed selection from 34 wpi to 59 wpi are consistent with previous observations that virus began to escape neutralization of the VRC26 lineage during this period [10]. The relaxed selection at this stage indicates an adaptive response of the VRC26 lineage to virus escape, which is consistent with the fact that cross-reactive neutralization breadth and potency developed during this period [10].

The measured selection pressure on the light chain CDRs of the VRC26 lineage shows positive or neutral selection from 34 wpi to 119 wpi and negative selection from 159 wpi to 206 wpi, which is consistent with that of heavy chain CDRs (Fig 6A). The observed positive selection at 38 wpi and 48 wpi may be the result of accommodating adaptive substitutions in the heavy chain such that antibody assembly and paraptope conformation can be stabilized. The FWRs of both heavy chain and light chain showed similar levels of negative selection at all time points.

Similar to the VRC26 lineage, the selection pressure on CDRs of the CH103 lineage heavy chain changed from positive selection to negative selection over time (Fig 6B). Consistent with the heavy chain CDRs, the selection pressure on the light chain CDRs changed from positive selection at 53 wpi to negative selection at 92 wpi. However, the selection pressure on the light chain CDRs changed to neutral at 144 wpi. The positive selection on the heavy and light chain CDRs at 53 wpi likely reflects viral escape around this period [11]. Since the neutralization of CH103 became broad around 78 wpi [11], the negative selection on the CDRs of both heavy and light chains at 92 wpi may reflect protection of the developed potency and breadth. However, since virus escaped CH103 neutralization around 144 wpi, it is reasonable to observe relaxed selection at this time point for the light chain CDRs. The FWRs of both heavy and light chains show negative selection across the study period. The strength of negative selection on the heavy chain FWRs did not change significantly over time. The longitudinal variations in selection pressure on the light chain FWRs are correlated with those of the CDRs, implying co-evolution of the CDRs and the FWRs.

For all clades of the VRC01 lineage, we observed significant negative selection on the CDRs of both heavy chain and light chain for most time points, with the exception of clade L3 (Fig 6C–6F). The negative selection strength on the CDRs is comparable to that on the FWRs, suggesting strong functional selection. This is potentially because VRC01 targets the conserved CD4 binding site in the Env protein, which evolves slowly. The serum IgG neutralization data showed that autologous viruses isolated from time points of 2001, 2006, and 2009 were resistant to neutralization by concurrent serum IgG antibodies [33], suggesting viral escape is frequent in the donor and may be the cause of the observed relaxation of negative selection of VRC01 clades. However, those neutralization results only reflected the evolution of a portion of the lineage members due to limited availability of antibody sequences at the time, and the evolutionary scenario may appear different when more lineage members are added to the analyses.

**Fig 7:**
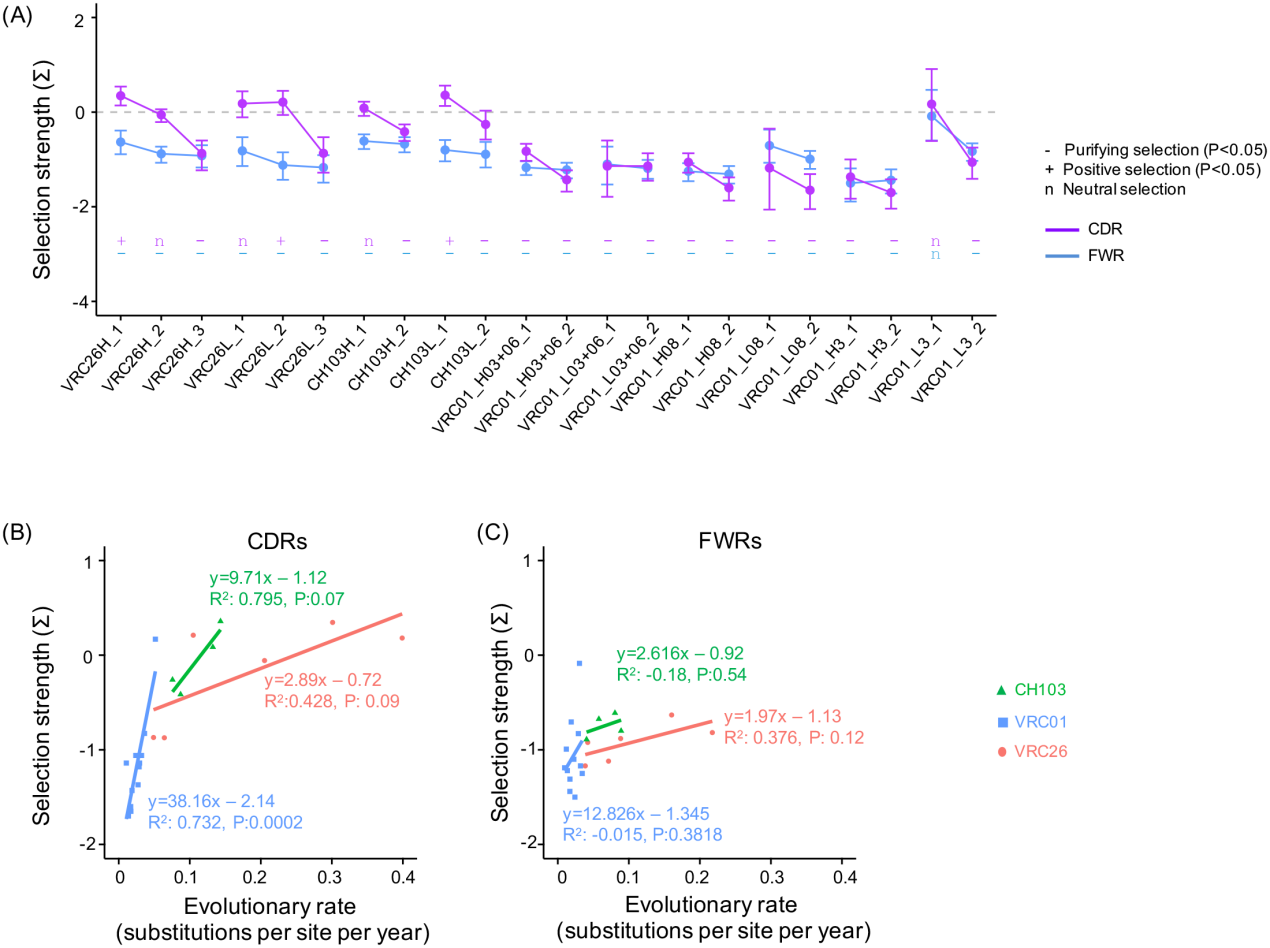
Selection pressure changes of the three lineages correlate with evolutionary rate decrease. (A) Estimated selection strength for the stages of the three lineages. For all three lineages, the negative selection strength of later stages is comparable or stronger than earlier stages. The statistical significance of the measured selection strength is shown on the bottom of the plot with ‘-’, ‘+’ and ‘n’ denoting negative selection, positive selection, and neutral selection, respectively. (B) The selection strength on CDRs showed trends of correlation with the slowing of the evolutionary rate. Only the linear correlation for the CDRs of the VRC01 lineage is statistically significant. (C) The correlations between selection strength and evolutionary rate of FWRs. There are trends of correlation but none is statistically significant.

The estimated selection pressure for stages of the three lineages show trends consistent with the above analyses (Fig 7A). The CDRs of the VRC26 and CH103 lineages show stronger negative selection at later stages. For the VRC01 lineage, only clade 03+06 and 08 heavy chain and clade L3 showed stronger negative selection at the second stage than the first stage. The negative selection on the FWRs of both heavy and light chains of the three lineages became stronger at later stage but the change is not significant (the 95% HPD overlap) except clade L3 of the VRC01 lineage.

To quantify the correlations between selection pressure and evolutionary rate, we used linear regression to estimate the intra-lineage correlations between mean selection strength and mean evolutionary rate (Fig 7B and 7C). The correlation for the CDRs and FWRs were estimated separately since they undergo different selection pressures. We observed trends of correlation between selection strength and evolutionary rates for both CDRs and FWRs, but only the selection pressure on the CDRs of the VRC01 lineage is significantly correlated with evolutionary rate change (P<0.05, Fig 7B). In addition to use randomly chosen sequences to estimate the selection pressure on each stage, we also estimated the selection strength at each time point and stage of the three lineages using all available NGS sequences (S5 and S6 Figs). The results are in good agreement with those from randomly chosen sequences, with the major difference being that correlation between the selection pressure on the CH103 CDRs and evolutionary rate change becomes statistically significant (P = 0.035, S6B Fig). However, the statistical tests are based on a small number of data points, and did not consider the uncertainty in the mean evolutionary rate and the mean selection strength.

The selection pressure change cannot fully explain the evolutionary rate decrease. This is supported by the fact that while the FWRs of some stages of the three lineages undergo similar levels of negative selection (selection strength about -0.8, Fig 7C), their evolutionary rates can be more than 5-fold different. Moreover, we estimated the evolutionary rates for the 1^st^+2^nd^ and 3^rd^ codon positions separately for the three lineages (S7 Fig). Since the substitutions at the 3^rd^ codon position are mostly synonymous, the selection pressure on the 3^rd^ codon position is weak. However, we observe significant decrease of evolutionary rate at the 3^rd^ codon position, supporting the idea that evolutionary rate decrease is not regulated by selection pressure alone.

In summary, the FWRs undergo stronger negative selection than do the CDRs in the VRC26 and CH103 lineages, just as the evolutionary rate in the FWRs is slower than that in the CDRs. When the selection pressure becomes comparable, the AMS model predicts the evolutionary rates of the CDRs and the FWRs should also become comparable. The observation that the evolutionary rates in the CDRs of the VRC01 clades are comparable to that in the FWRs is consistent with this prediction. We do observe a trend of correlation between dynamics of selection pressure and evolutionary rate especially for CDRs, but the selection pressure alone cannot fully explain the evolutionary rate decrease.

### Longitudinal mutability dynamics of immunoglobulin genes

To examine the contribution of changes in mutability to evolutionary rate decrease, we predicted longitudinal mutability dynamics for CDRs and FWRs of the three antibody lineages based on the presence of AID hotspot and coldspot motifs. Briefly, we first estimated the mean mutational potency for each position of an immunoglobulin sequence, which is the normalized frequency of mutation for a nucleotide using the S5F model [34]. The mutability of a sequence is estimated by averaging over the mutational potency of all positions in both the forward and reverse strands and the mean mutability of a time point was calculated by averaging over the mean mutabilities of each sequence in a dataset. To compare mutability changes from ancestor sequences, we calculated the mutability for the inferred UCA/MRCAs of each of the three lineages. To quantify the correlation between predicted mutability and evolutionary rate change, we further predicted the mutability for each stage of the three lineages, and used linear regression to estimate the correlation coefficients between mutability and evolutionary rate.

**Fig 8:**
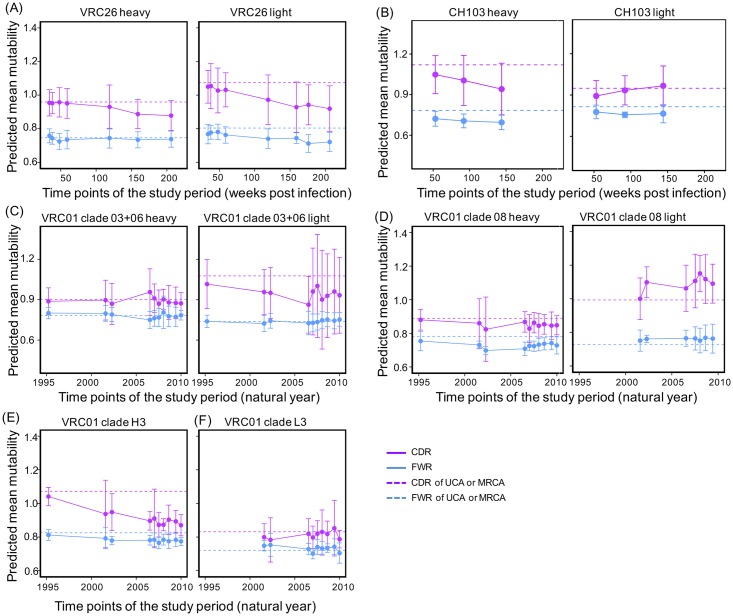
Dynamics of the predicted mutability for all time points of the three lineages. (A) Changes of predicted mutability of the VRC26 heavy (left) and light (right) chains. (B) Changes of predicted mutability of the CH103 heavy (left) and light (right) chains. (C) Changes of predicted mutability of the VRC01 clade 03+06 heavy (left) and light (right) chains. (D) Changes of predicted mutability of the VRC01 clade 08 heavy (left) and light (right) chains. (E) Changes of predicted mutability of the VRC01 clade H3. (F) Changes of predicted mutability of the VRC01 clade L3. Dashed line represents the mutability of UCA (VRC26 and CH103) or MRCA (VRC01 clades) of the lineages.

The results show that the 95% confidence interval for the mutability of the CDRs is wider than that of the FWRs, suggesting more variations in the mutability of the CDRs than in the FWRs (Fig 8). The mutability of the CDRs of both chains of the VRC26 lineage decreased starting at 119 wpi (Fig 8A). The mean mutability of the CDRs of the CH103 heavy chain decreased, but the mutability of the light chain CDRs was mostly unchanged (Fig 8B). The VRC01 lineage showed consistent mutability over the course of the study period, except for clade H3, the mutability of which decreased over time (Fig 8C–8F). Overall, the mutability of the FWRs showed variations but no consistent trend of decrease over time for both heavy and light chains of the three lineages with two exceptions (CH103 heavy chain and VRC26 light chain).

The changes in mutability of the three lineages varied with respect to the mutability found in the UCA or MRCA (Fig 8). The mean mutability of the CDRs of the VRC26-lineage heavy chain is similar to that of the UCA at early time points, but slightly lower than the UCA by the end of the study period, while the mutability of the FWRs remains comparable to that of the UCA (Fig 8A, left). The mean mutability of both the CDRs and the FWRs of the VRC26-lineage light chains are lower than that of the UCAs (Fig 8A, right). For the CH103 lineage, the mutability of both CDRs and FWRs of heavy chain is lower than the UCA (Fig 8B, left). However, the mutability of the light chain CDRs increased to a similar level as the UCA at 92 wpi and 144 wpi (Fig 8B, right). For the VRC01 lineage clades (Fig 8C–8F), the mutability of the FWRs of both heavy and light chains are comparable to those of the inferred MRCAs, while the mutability of the CDRs are comparable to the MRCAs except for clade 08 heavy chain, which showed lower mutability.

**Fig 9:**
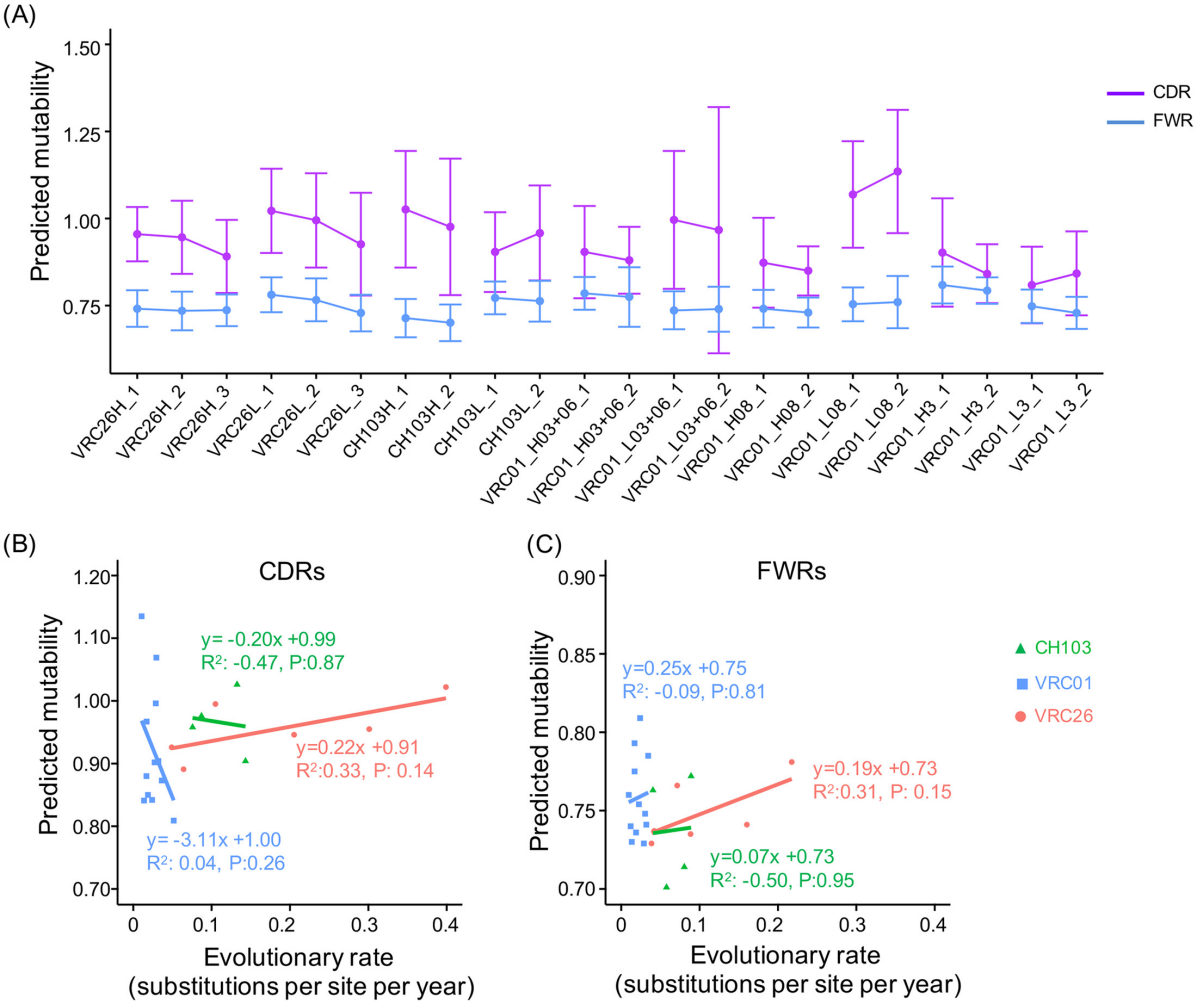
The longitudinal changes of the predicted mutability correlate weakly with evolutionary rate decrease. (A) Predicted mutability for the stages of the three lineages. (B) The correlations between predicted mutability and evolutionary rate of CDRs were estimated using linear regression. No statistically significant correlation between the selection strength and evolutionary rate was observed. (C) The linear correlations between predicted mutability and evolutionary rate of FWRs. No significant correlation was observed for the three lineages.

Consistent with the above analyses for time points, we found similar trends of mutability changes for the stages of the three lineages (Fig 9). To quantify the correlations between the predicted mutability and evolutionary rate, we used linear regression to measure the correlations between the mean mutability and the mean evolutionary rate for the CDRs and FWRs separately for each lineage (Fig 9B and 9C). Only the CDRs and FWRs of the VRC26 lineage show a trend of meaningful correlation, but even these do not reach the level of significance.

In summary, a longitudinal decrease in mutability is not consistently observed, indicating that while changes in mutability may contribute to evolutionary rate change, it is not the dominant regulator. However, the mutability is predicted using S5F model, which is trained using a limited set of heavy chain repertoire sequences and may not fully incorporate the context dependency of the AID hot-spots [34]. It is likely that there is uncertainty in the predicted mutability changes, which is not incorporated in our analyses. Thus, the correlation between antibody sequence mutability change and evolutionary rate requires further investigations.

### Combined effects of selection pressure and mutability on evolutionary rate decrease

We used multiple regression to evaluate the combined effects of selection pressure and mutability on the evolutionary rate decrease for the CDRs and FWRs of each lineage. The fitted R^2^ showed that the two factors can explain about 70% of the variations of the evolutionary rate changes except the CDRs of the VRC26 lineage and the FWRs of the VRC01 lineage (S2 Table), suggesting dominant contributions to evolutionary rate changes. However, except for the CDRs of the VRC01 lineage, the examined correlations are not statistically significant, consistent with the linear regression analysis. This may be because we tested the correlations on a small sample size. Moreover, we cannot rule out the possibility that the correlations between evolutionary rate and the two factors are non-linear. For example, if the data points of the three lineages are combined, the apparent correlation between evolutionary rate and selection pressure may be non-linear. However, we did not do this because we think the evolution of each lineage may be differently driven by virus evolution and other factors described below in the Discussion section. This is consistent with the fact that even the clades of the VRC01 lineage showed some variations of mutability change. Thus, further investigations with more antibody lineages and a more sophisticated model will be required to verify the conclusions here.

## Discussion

Epitope change is an important mechanism used by pathogens to escape antibody neutralization, raising the question of how quickly antibodies can gain affinity via somatic hypermutation. The available longitudinal data of broadly HIV-1 neutralizing antibody lineages provides the opportunity to shed light on this question. In this study, we measured the longitudinal dynamics of evolutionary rates of antibody lineages whose developments were followed for more than 2 years. Our results show that the evolution of antibody variable regions, and especially the CDRs, can be 20-fold faster than that of the HIV-1 in the first few weeks post lineage initiation, providing a repertoire of paratope variants for epitope-directed selection. We also showed that CDRs evolve about 1.5–2 fold faster than FWRs during the early stages of antibody evolution, due to both stronger selection pressure and lower mutability in FWRs. The observed antibody evolutionary rate dynamics provide useful information for antibody elicitation during vaccination. One interesting question not answered here is whether antibody maturation following vaccination can reach an evolutionary rate as high as that achieved during viral infection.

Longitudinally, the highest evolutionary rates are not sustained throughout antibody maturation. The evolutionary rates of the three bnAb antibody lineages examined here consistently decreased, suggesting common mechanisms regulating antibody evolution. Because the three lineages are from different donors and V(D)J recombinations, the changes in evolutionary rate change appear to be independent of these factors. Our analyses of the dynamics of functional selection and mutability showed that neither could fully explain the dramatic evolutionary rate decrease. Although the observed trends of changes of selection pressure and evolutionary rate are consistent, the limited amount of data and lack of statistical significance makes it impossible to draw firm conclusions. Moreover, these factors cannot explain why the evolutionary rate of the 3^rd^ codon position also consistently decreased in these three lineages. One possibility is that the measured selection strength is affected by evolutionary rate changes. For example, it is possible that when the evolutionary rate is high, more passenger mutations (neutral or slightly detrimental) could become fixed in the B cell lineage than when evolutionary rate is low. Further investigations are required to more accurately estimate selection pressure, evolutionary rates, and mutability.

An additional possible mechanism for the observed evolutionary rate change is a decrease in the rate of mutations generated by AID modulated by mechanisms other than antibody sequence mutability. Although memory B cells can undergo additional SHM upon returning to a germinal center [35], it is unclear whether the rate of mutation remains constant. Lowering the mutation rate can be beneficial evolutionarily, because most mutations are likely to decrease the antigen-paratope interaction once affinity maturation has occurred. Thus, a decrease in mutation rate (and hence evolutionary rate) may be a mechanism to protect immune memory. Previous studies of memory B cells have revealed very low frequencies of SHM during secondary GC reactions [29, 36]. This change could be regulated via functional regulation of the SHM machinery, or other unknown mechanisms. Evidence supporting the first possibility is that FWRs are less mutated than CDRs due to having fewer AID hotspots [37, 38]. However, we did not observe a consistent decrease in AID hotspots during antibody evolution, as reflected in the mean mutability of the sequences. Nonetheless, the biochemical mechanism of hot-spot motif recognition by hypermutation machinery in B cells is still obscure. If mutation rate is severely decreased during evolution of a B cell lineage via functional regulation of the hypermutation machinery, the regulation is probably achieved via genetic or epigenetic means, which can easily transmit the information to progeny B cells. Further comparisons of the mutation rates in primary and secondary GC reactions would be required to confirm this hypothesis.

Another hypothesis is that antibody evolution is modulated via either direct or indirect regulation of the frequency of B cell proliferation. Due to strong selection pressure from the host immune system, HIV-1 viral escape occurs with high frequency [10, 11, 33]. It is likely that the memory B cells of a lineage would have less and less chance to be activated as escape mutations spread through the viral population. Weak binding affinity of germinal center B cells to evolved epitope variants could also slow B cell proliferation. Furthermore, multiple antibody lineages are elicited in a patient during HIV-1 infection. The competition between lineages [4, 39], targeting either the same or heterologous antigens within a germinal center, may slow the proliferation and evolution of B cell lineages with low binding affinity. In addition, secreted antibodies circulating in the blood can visit germinal center and compete with GC B cells for antigen binding [40]. Thus, the mean number of substitutions observed at later time points would be fewer if a B cell lineage fails to acquire antigen under competitive pressure.

The observed evolutionary rate decrease could also be a HIV-1- or chronic-infection-specific phenomenon. This is because HIV-1 infection can destroy GCs via depletion of infected Tfh cells and B cells, and disruption of the FDC network [17, 41, 42]. Thus, fewer B cells survive and proliferate at later stages of infection when anti-HIV-1-specific Tfh cells and GC become fewer. In addition, many other viruses evolve in their hosts an order of magnitude slower than HIV-1 [43], meaning the frequency of viral escape is low and the AMS model may be more effective at describing the regulation of antibody evolution. Another possibility is that detrimental mutations introduced by AID accumulate in the genome outside the Ig locus during cycles of proliferation [44], so that the chance of B cells survival becomes low.

To further explore the mechanisms underlying antibody evolution, more characterization of the developmental history of single B cell lineages is required. For example, fluorescent labeling techniques allow the estimation of a B cell’s proliferation history [1]. Combined with high-throughput single cell sequencing, in principle the correlation of rate changes with genetic and epigenetic changes of the B cell could be explored. As technology advances, especially *in vivo* B cell imaging [4], it may also be possible to understand the dynamics of memory B cell reentry into GCs, and proliferation under chronic epitope variation.

## Materials and Methods

### Sequence datasets

The curated sequence datasets of the CH103 lineage were retrieved from NCBI GenBank database (Accession Number: KC575845-KC576303 for heavy chain and KC576304-KC576477 for light chain) [11]. The curated antibody sequences of the VRC26 lineage were from [10, 20, 26] (GenBank Accession Number: KJ134860-KJ134387, KT-371118-KT371169 for VRC26 heavy chain, KJ134388-KJ134859, KT371170-KT371320 for VRC26 light chain, and KT371076-KT371117 for heavy and light chain sequences of 159 and 193 wpi). The VRC01 lineage sequences were from [16] (GenBank Accession Number: KP840719-KP841751 for VRC01 heavy chain, and KP841752-KP842237 for VRC01 light chain).

### Sequence divergence from UCA or MRCA and substitution saturation test

Sequences of each dataset were aligned using ClustalO (version: 1.2.0) [45]. The substitution saturation tests for all datasets were performed using the substitution index method in DAMBE5 (version: 6.1.7) [22, 23]. The UCAs of the VRC26 and CH103 lineages were from previous studies [10, 11]. The MRCAs of the clades of the VRC01 lineage were inferred using the Maximum Likelihood method in MEGA6 (build number: 6140220) [21], with the GTR+ Γ substitution model and gamma distribution used to measure genetic distance and estimate substitution rate variations among sites respectively.

### Evolutionary rate calculation

To increase the ‘clock-like-ness’ of the dataset [19, 46], at most 20 sequences were randomly selected from each time point for rate calculation. If a time point has less than 5 sequences, the time point was removed. Evolutionary rate was estimated using BEAST2 (version v2.2.1) [24]. The GTR+Γ substitution model was used to estimate genetic distance between sequences and modulate substitution rate heterogeneity among sites respectively. The relaxed log-normal clock model was used to estimate the rate variations among branches. The coalescent Bayesian Skyline population model was used as tree prior [47]. For each dataset, the Monte Carlo Markov Chain (MCMC) simulations were ran until convergence and parameters were sampled with effective sample size larger than 200 [24].

For all simulations of the CH103 lineage, the most recent common ancestors (MRCAs) or the roots of the time scaled Bayesian trees were assumed to appear one week post virus infection (wpi) or later [11] and sampled from a uniform distribution of [1 wpi, +∞). Previous studies showed that the VRC26 lineage was probably initiated around 30 wpi [10]. Thus, for all BEAST2 MCMC simulations of the 2^nd^ and 3^rd^ stages of the VRC26 lineage, the roots of the time scaled Bayesian trees were sampled from a uniform distribution of [30 wpi, +∞). However, the roots of the time scaled Bayesian trees for the 1^st^ stage were sampled from a uniform distribution of [23 wpi, +∞), rather than [30 wpi, +∞), due to the fact that 30 wpi is too strong a constraint on the height of the time scaled Bayesian trees and prevents the simulation from initiating. Thus, the mean evolutionary rate of the 1^st^ stage is underestimated. To calculate the evolutionary rates for the complement determining regions (CDRs) and framework regions (FWRs), the BEAST2 simulation for a dataset was ran with three partitions: the V(D)J region sequences, the CDR region sequences, and the framework region sequences. For each simulation step, the V(D)J region sequences were used to construct a phylogenetic tree and estimate substitution model parameters. Then the evolutionary rates for the CDRs and FWRs were calculated using the estimated parameters. The same method was used to estimate the evolutionary rates for the 1^st^+2^nd^ and 3^rd^ codon positions of a dataset. The boundaries of CDRs and FWRs were predicted using IMGT server (http://www.imgt.org/IMGT_vquest/share/textes/) [48].

To examine the effects of sample size on the estimated evolutionary rates, we calculated the evolutionary rates for the 2^nd^ stage of the VRC26 and CH103 heavy chains with three sample sizes (30, 20, and 10 randomly selected sequences per time point). The results showed that the estimated evolutionary rates are robust with respect to sample size changes (S2A Fig). We also used the random local clock model to estimate evolutionary rates for the VRC26 heavy chain and simulated dataset with decreasing evolutionary rate (See below). However, the simulation failed to converge to reasonable posterior probability values (reported log likelihood values greater than 0) (S1 Fig), possibly because the simulation algorithm was unable to find optimal tree structures for our datasets. Our simulations further showed that the estimated evolutionary rates for the 2^nd^ stages of the VRC26 and CH103 heavy chains were consistent when different tree priors (constant population size or Bayesian Skyline) were used (S2B Fig).

### Validation of the time-bin method and stage method for characterizing evolutionary rate change

We first simulated the evolution of a gene with a constant evolutionary rate using TreesimJ [49]. The simulation was initiated from a randomly generated ancestor sequence with 450 nucleotides. The mutation rate was set to 0.0001 substitutions per site per generation and Jukes-Cantor substitution model was used for modeling nucleotide substitution bias. The ratio of non-synonymous/synonymous substitution was set to 0.007. A constant population size (1000) was used during the simulation. The simulation was run for 12,000 generations, and 40 DNA sequences were sampled every 10 generations.

We then selected sequences from 8 generations (480^th^, 960^th^, 1320^th^, 1680^th^, 1920^th^, 2160^th^, 2280^th^, and 2400^th^) to estimate the evolutionary rate of the dataset (CR dataset) using BEAST2 with both restricted molecular clock model and relaxed log-normal clock model, giving a base evolutionary rate of α. We then built phylogenetic tree for the simulated dataset using MEGA6 [21] and measured the tree shape imbalance using the colless method in apTreeshape (S3 Fig). The analysis showed the tree shape of the CR dataset (the colless index with PDA and Yule normalizations are 1.35 and 19.50 respectively) is similar to that of antibody phylogenetic tree (the colless index with PDA and Yule normalizations are 1.26 and 15.68 respectively for VRC26 heavy chain) [50]. We then estimated evolutionary rate changes over time for the CR dataset (S4 Fig). As expected, both methods showed no changes of evolutionary rate, suggesting the two methods are comparable for estimating evolutionary rate when no rate changes over time.

To generate a data set with decreasing evolutionary rate (DR dataset), we changed the generation labels of the sequences of the above time points to 120, 240, 360, 480, 600, 720, 840, and 960 respectively. The time intervals between sequential time points were 25%, 33%, 33%, 50%, 50%, 100%, and 100% of the original time intervals respectively. Correspondingly, the evolutionary rates between sequential time points were expected to be 4α, 3α, 3α, 2α, 2α, α, and α. In addition, the time interval from the root or MRCA to the 120^th^ generation is 25% of the original time interval (480 generations). Therefore, the evolutionary rate from the MRCA to the 120^th^ generation became 4α. We then estimated the evolutionary rate for the dataset using BEAST2 with coalescent constant population tree prior, Jukes-Cantor substitution model, and relaxed log-normal clock model. The MRCA of the CR dataset estimated by both time-bin and stage method is 110^th^ generation, we therefore set the MRCA of the DR dataset to 27.5 generation (four times shorter than the CR dataset) for simulation. A maximum clade credibility (MCC) tree was constructed using TreeAnnotator v2.2.1 in the BEAST2 package [24]. The time scale of the MCC tree was then discretized into 8 bins (MRCA to 120^th^, 120^th^ to 240^th^, 240^th^ to 360^th^, 360^th^ to 480^th^, 480^th^ to 600^th^, 600^th^ to 720^th^, 720^th^ to 840^th^, and 840^th^ to 960^th^ generations), and the mean evolutionary rate for each bin was calculated by averaging over the evolutionary rates of all branches within the time bin using the following formula [24]:

$$\mu \;=\;\Sigma {(r}_{i}*t_{i})/\Sigma t_{i}$$ (1)

where *μ* is the mean rate of the time bin; r_i_ is the rate of branch i; and t_i_ is the length of time branch i which is within the time bin.

Because the 95% highest posterior density interval (HPD) of *μ* cannot be estimated from a single tree, we used another method to estimate the evolutionary rates of the 8 time bins. Briefly, 1000 time scaled Bayesian trees with high posterior probabilities were retrieved from the MCMC simulation. For each time scaled Bayesian tree, we estimated *μ* for the 8 time bins as described above. Then, the mean and 95% HPD of *μ* were calculated for each time bin from the 1000 estimates, using methods similar to BEAST2 [24].

To verify the stage method, the 8 time points were separated into three stages: 120^th^ to 480^th^, 360^th^ to 720^th^, and 600^th^ to 960^th^ generations. The mean evolutionary rate for each stage was estimated using BEAST2 with coalescent constant population tree prior, Jukes-Cantor substitution model, and relaxed log-normal clock model [24]. By using formula (1), we estimated the expected mean evolutionary rate of the three stages. For example, for stage 1, the evolutionary rates of the MRCA to 240^th^ generation and the 240^th^ to 480^th^ generation are 4α and 3α respectively (see above). The expected evolutionary rate of stage 1 is (4α*120 + 3α*120)/240 = 3.5α. Similarly, the expected evolutionary rates of the later two stages are 2.5α and 1.5α respectively.

### Selection pressure estimation

To estimate selection pressure for a time point of a dataset and to remove selection signals from earlier time points, we first built a phylogenetic tree using all available sequences of all time points for each chain of a lineage. Then we inferred the sequences for all internal nodes using MEGA6 (maximum likelihood method and GTR+Γ substitution model) [21]. Starting from a selected terminal branch, we searched for the first internal node which also gave rise to a terminal branch from an earlier time point. The sequence of the internal node was then used as reference to remove substitutions evolved at earlier time points. BASELINe v1.3 was used to estimate the selection strength for each selected sequence of a time point and for a time point from selected sequences [27, 28, 51]. To be consistent with the evolutionary rate analyses, we first estimated the selection strength for each time point of each lineage and chain using the same sequences used for evolutionary rate calculation. We then used all available sequences of a time point to estimate the selection strength, and the measured selection strength for most time points of the three lineages were in good agreement with those estimated using randomly selected sequences (Figs 6 and S5). We then estimated the selection strength for each stage of a lineage chain using both the randomly selected dataset and all available sequences, the results of which were consistent (Figs 7A and S6). To quantify the correlation between selection strength and evolutionary rate, we used linear regression in R to estimate the intra-lineage correlation coefficients between evolutionary rate and selection strength for CDRs and FWRs respectively.

### Mutability estimation

A sliding window of five nucleotides was used to calculate the mutability of the central nucleotide. The mutability values of the motifs were from the S5F antibody-specific substitution model [34]. The mean mutability of a sequence was calculated by averaging over the mutability of all positions in both the forward and reverse strands [52]. The mutability for a stage of a chain was calculated using the mean mutability of the selected sequences of the stage. The linear regression fitting of the correlation between evolutionary rate and mutability was performed in R.

## Supporting Information

S1 Table No significant substitution saturation observed for the datasets used in this study. The substitution saturation test for each dataset was performed using DAMBE5. DAMBE5 examines whether the measured substitution saturation index (I_ss_) of a dataset is significantly different from the theoretical I_ss_. The theoretical I_ss_ is estimated assuming the underlying tree structure of a dataset is either symmetric (I_ss_.cSym) or asymmetric (I_ss_.cAsym). The analysis showed our datasets were far away from significant substitution saturation under both assumptions (P<0.05).
(TIF)

S2 Table Combined effects of selection pressure and mutability on evolutionary rate changes using multiple linear regression. The measured effects of selection pressure and mutability on evolutionary rate changes are consistent with the linear regression analyses (Figs 7 and 9). Briefly, the R-square showed a large portion of the evolutionary rate changes could be explained by selection pressure change and mutability change except VRC26 CDRs and VRC01 FWRs. But only the correlation between the selection pressure and the evolutionary rate of VRC01 CDRs is statistically significant. The results should be interpreted with caution since the test was performed on a limited dataset and the uncertainty of the measured selection strength and evolutionary rate were excluded.
(TIF)

S1 Fig Posterior probability distribution of BEAST2 simulations with local random molecular clock model and relaxed log-normal clock. (A) The posterior distribution of a BEAST2 simulation using the simulated dataset with decreasing evolutionary rate (eight time points, see Methods). (B) The posterior distribution of a BEAST2 simulation using the VRC26 lineage heavy chain sequences of all time points. For both simulations, the posterior probability failed to converge to reasonable values, suggesting the local random clock model cannot be applied to our datasets. The distribution for simulations with local random clock and relaxed log-normal clock were colored black and green respectively.
(TIF)

S2 Fig Sample size and coalescent tree priors have no effect on the estimation of evolutionary rates of datasets in this study. (A) The effect of sample size on evolutionary rate estimation. We randomly sampled 30, 20, and 10 sequences from each time point to estimate the evolutionary rates for the second stages of the VRC26 and the CH103 heavy chains with coalescent Bayesian Skyline population tree prior. The sample sizes showed little effect on the estimated evolutionary rate. (B) The effect of tree priors on evolutionary rate estimation. The evolutionary rates of the same two datasets (20 sequences per time point) in (A) were estimated using the constant coalescent population tree prior (labels end with ‘C’) and coalescent Bayesian skyline population tree prior (labels end with ‘B’) respectively. The estimated evolutionary rates for the same dataset are consistent, suggesting the two types of tree priors have little effect on the estimation of evolutionary rate of our datasets.
(TIF)

S3 Fig Phylogenetic trees of the VRC26 heavy chain and simulated dataset. The simulated dataset (B) showed (the colless index with PDA and Yule normalizations are 1.35 and 19.50 respectively) is similar to that of the VRC26 heavy chain phylogenetic tree (A, the colless index with PDA and Yule normalizations are 1.26 and 15.68 respectively).
(TIF)

S4 Fig Evolutionary rate changes for the CR dataset estimated using time-bin and stage methods are comparable. Compared to expected evolutionary rate (red), both time-bin (A, gray) and stage (B, blue) methods showed no evolutionary rate changes for the CR dataset. This suggests the two methods are comparable when no rate changes over time.
(TIF)

S5 Fig Selection pressure of time points of the three lineages measured using all available sequences. The results are consistent with that of Fig 6. (A) Selection pressure changes of the VRC26 lineage heavy (left) and light (right) chains. (B) Selection pressure changes of the CH103 lineage heavy (left) and light (right) chains. (C) Selection pressure changes of the VRC01 clade 03+06 heavy (left) and light (right) chains. (D) Selection pressure changes of the VRC01 clade 08 heavy (left) and light (right) chains. (E) Selection pressure changes of the VRC01 clade H3. (F) Selection pressure changes of the VRC01 clade L3.
(TIF)

S6 Fig Selection pressure of stages of the three lineages measured using all available sequences and the correlations between selection pressure and evolutionary rate changes. (A) Selection pressure changes over time. (B) Linear correlations between selection pressure and evolutionary rate of CDRs. (C) Linear correlations between selection pressure and evolutionary rate of FWRs.
(TIF)

S7 Fig The estimated evolutionary rates for the 1^st^+2^nd^ and the 3^rd^ codon positions of the three lineages decrease over time. (A) The evolutionary rate changes for the 1^st^+2^nd^ and the 3^rd^ codon positions of the VRC26 lineage heavy (left) and light (right) chains. (B) The evolutionary rate changes for the 1^st^+2^nd^ and the 3^rd^ codon positions of the CH103 lineage heavy (left) and light (right) chains. (C) The evolutionary rate changes for the 1^st^+2^nd^ and the 3^rd^ codon positions of the VRC01 clade 03+06 heavy (left) and light (right) chains. (D) The evolutionary rate changes for the 1^st^+2^nd^ and the 3^rd^ codon positions of the VRC01 clade 08 heavy (left) and light (right) chains. (E) The evolutionary rate changes for the 1^st^+2^nd^ and the 3^rd^ codon positions of the VRC01 clade H3. (F) The evolutionary rate changes for the 1^st^+2^nd^ and the 3^rd^ codon positions of the VRC01 clade L3. The evolutionary rates of both the 1^st^+2^nd^ and the 3^rd^ codon positions decreased over time, except clade H3, L3, and the 3^rd^ codon positions of two clades (03+06 light chain and both heavy and light chains of clade 08) in the VRC01 lineage. This suggests evolutionary rate is systematically regulated and selection pressure change cannot fully explain the slowing of evolutionary rate.
(TIF)
