# Correction: Effects of Darwinian Selection and Mutability on Rate of Broadly Neutralizing Antibody Evolution during HIV-1 Infection

**DOI:** 10.1371/journal.pcbi.1007138

**Published:** 2019-06-14

**Authors:** The PLOS Computational Biology Staff

## Notice of republication

This article was republished on August 11, 2016, to correct an error with Fig 4 that was introduced during the typesetting process. The publisher apologizes for the errors. Please download this article again to view the correct version.

## Supporting information

S1 File Republished, corrected article. (PDF)
