# Supplementary figures and images for: Effects of Darwinian Selection and Mutability on Rate of Broadly Neutralizing Antibody Evolution during HIV-1 Infection
Source: PLoS Comput Biol. 2016 May 18;12(5):e1004940. doi: 10.1371/journal.pcbi.1004940 (PMC4871536; doi:10.1371/journal.pcbi.1004940)

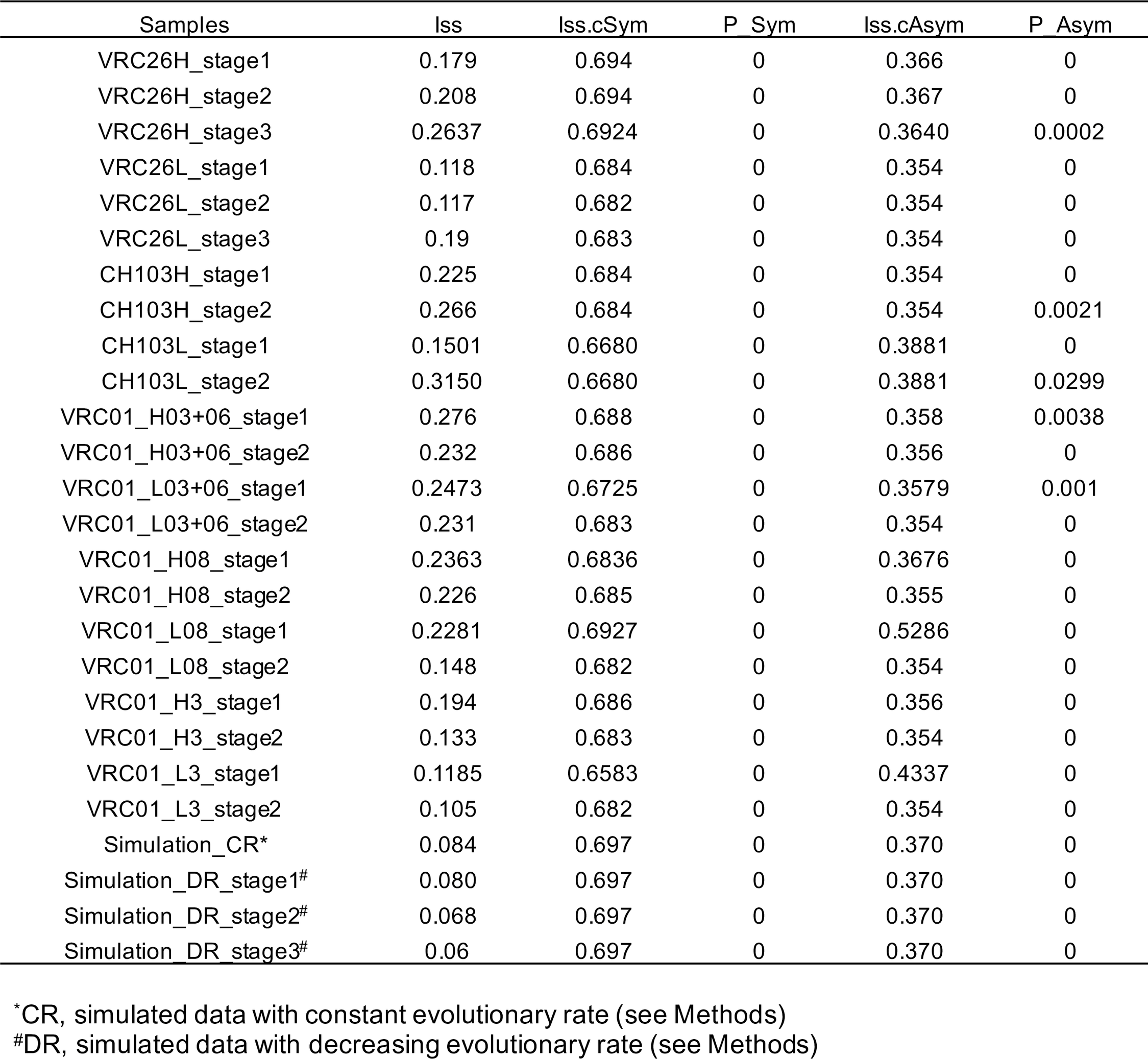

Supplement: S1 Table — The substitution saturation test for each dataset was performed using DAMBE5. DAMBE5 examines whether the measured substitution saturation index (Iss) of a dataset is significantly different from the theoretical Iss. The theoretical Iss is estimated assuming the underlying tree structure of a dataset is either symmetric (Iss.cSym) or asymmetric (Iss.cAsym). The analysis showed our datasets were far away from significant substitution saturation under both assumptions (P<0.05). (TIF) [file pcbi.1004940.s001.tif]

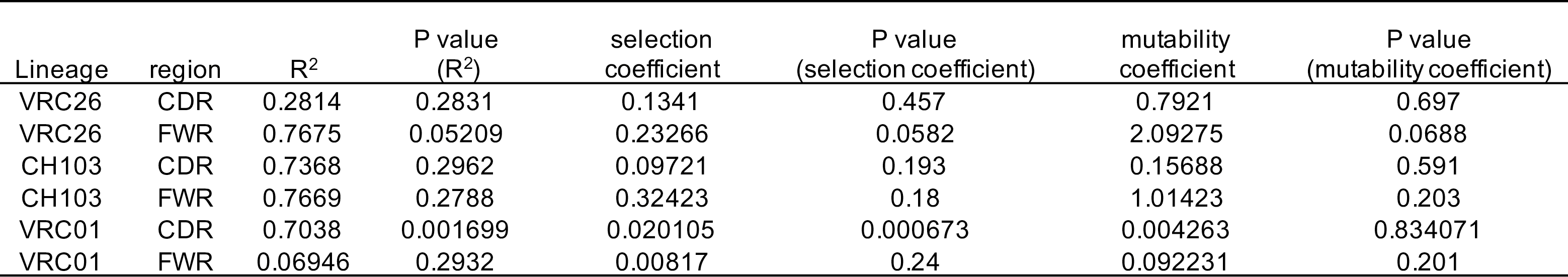

Supplement: S2 Table — The measured effects of selection pressure and mutability on evolutionary rate changes are consistent with the linear regression analyses (Figs 7 and 9). Briefly, the R-square showed a large portion of the evolutionary rate changes could be explained by selection pressure change and mutability change except VRC26 CDRs and VRC01 FWRs. But only the correlation between the selection pressure and the evolutionary rate of VRC01 CDRs is statistically significant. The results should be interpreted with caution since the test was performed on a limited dataset and the uncertainty of the measured selection strength and evolutionary rate were excluded. (TIF) [file pcbi.1004940.s002.tif]

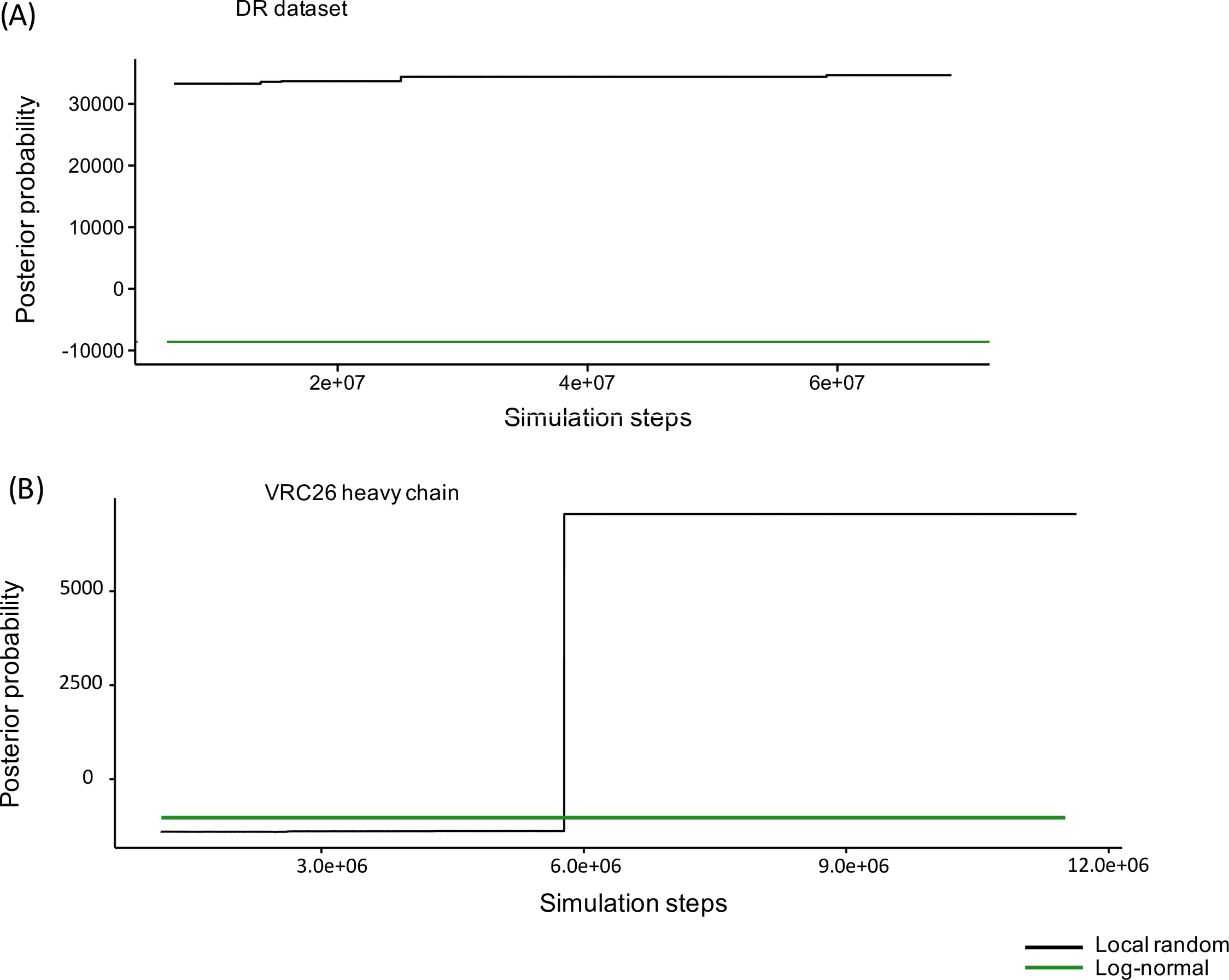

Supplement: S1 Fig — (A) The posterior distribution of a BEAST2 simulation using the simulated dataset with decreasing evolutionary rate (eight time points, see Methods). (B) The posterior distribution of a BEAST2 simulation using the VRC26 lineage heavy chain sequences of all time points. For both simulations, the posterior probability failed to converge to reasonable values, suggesting the local random clock model cannot be applied to our datasets. The distribution for simulations with local random clock and relaxed log-normal clock were colored black and green respectively. (TIF) [file pcbi.1004940.s003.tif]

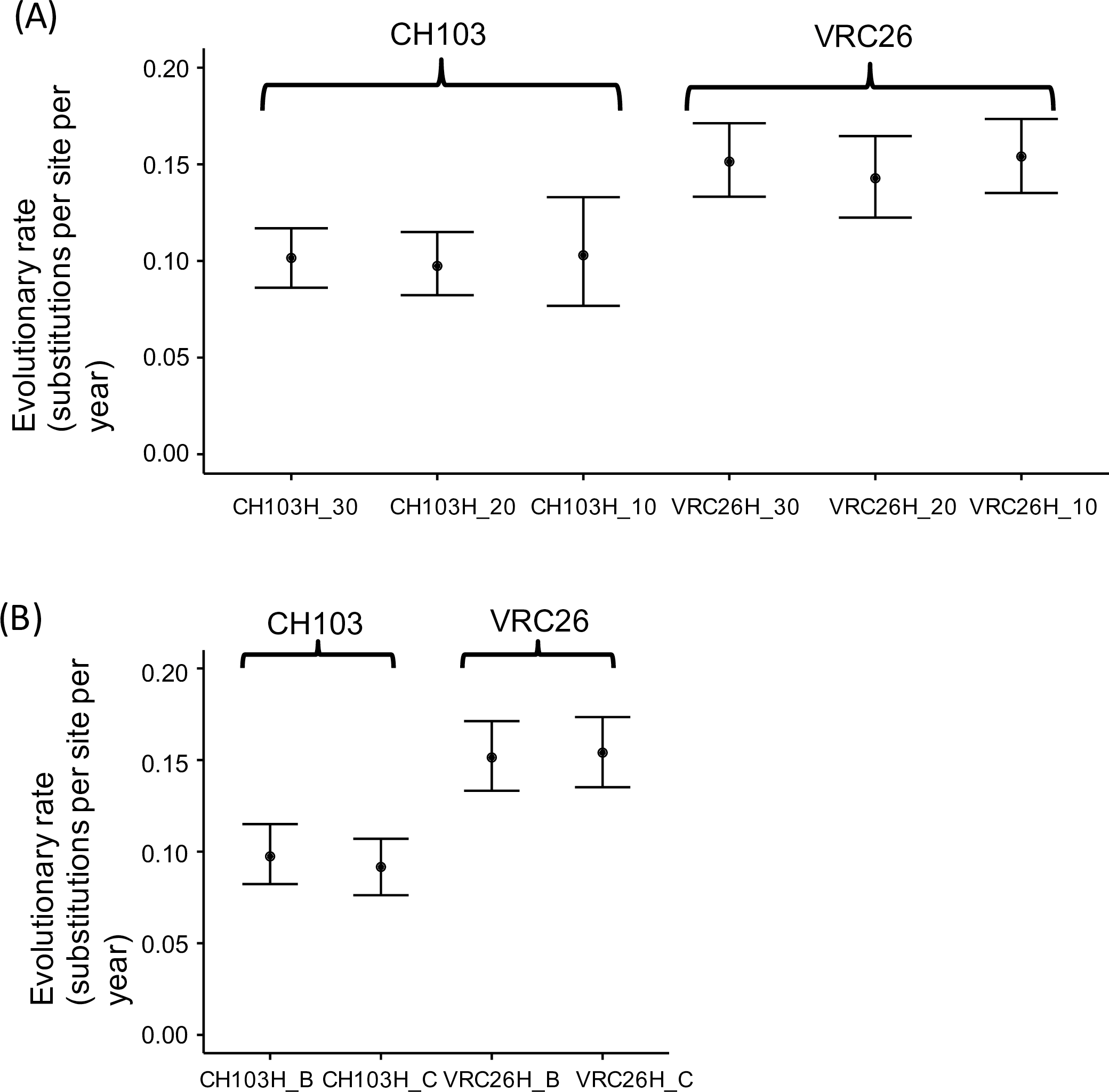

Supplement: S2 Fig — (A) The effect of sample size on evolutionary rate estimation. We randomly sampled 30, 20, and 10 sequences from each time point to estimate the evolutionary rates for the second stages of the VRC26 and the CH103 heavy chains with coalescent Bayesian Skyline population tree prior. The sample sizes showed little effect on the estimated evolutionary rate. (B) The effect of tree priors on evolutionary rate estimation. The evolutionary rates of the same two datasets (20 sequences per time point) in (A) were estimated using the constant coalescent population tree prior (labels end with ‘C’) and coalescent Bayesian skyline population tree prior (labels end with ‘B’) respectively. The estimated evolutionary rates for the same dataset are consistent, suggesting the two types of tree priors have little effect on the estimation of evolutionary rate of our datasets. (TIF) [file pcbi.1004940.s004.tif]

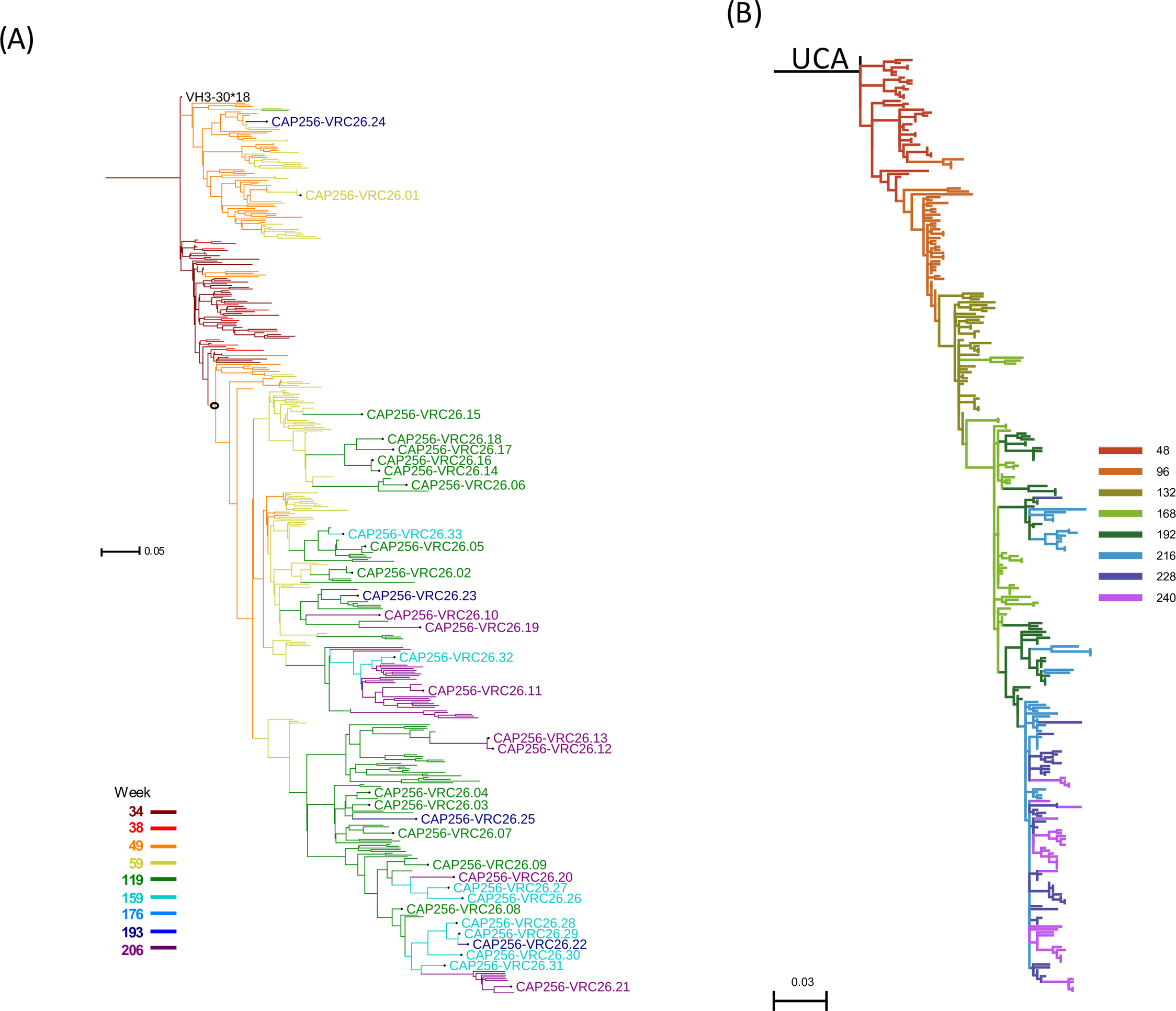

Supplement: S3 Fig — The simulated dataset (B) showed (the colless index with PDA and Yule normalizations are 1.35 and 19.50 respectively) is similar to that of the VRC26 heavy chain phylogenetic tree (A, the colless index with PDA and Yule normalizations are 1.26 and 15.68 respectively). (TIF) [file pcbi.1004940.s005.tif]

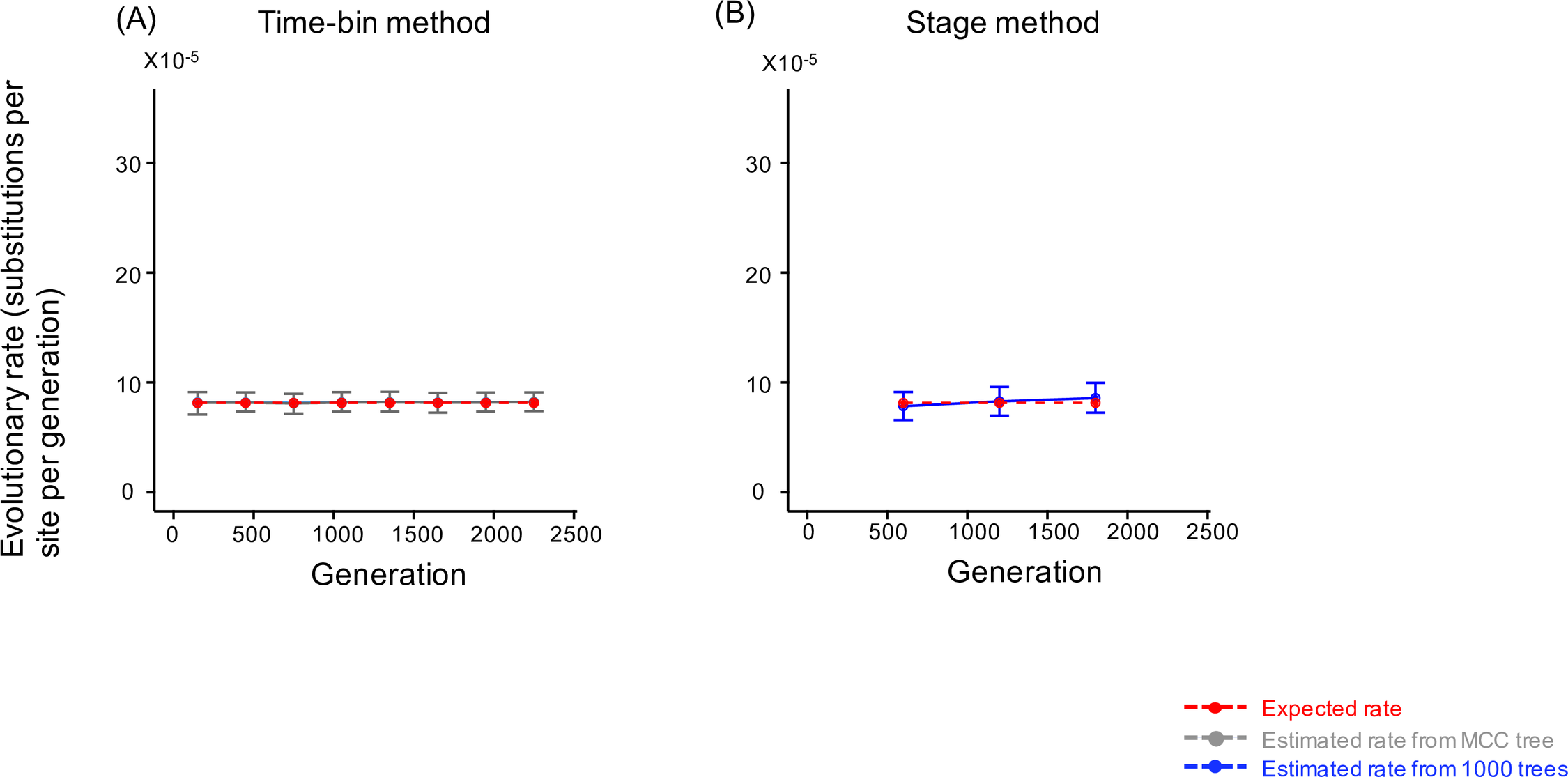

Supplement: S4 Fig — Compared to expected evolutionary rate (red), both time-bin (A, gray) and stage (B, blue) methods showed no evolutionary rate changes for the CR dataset. This suggests the two methods are comparable when no rate changes over time. (TIF) [file pcbi.1004940.s006.tif]

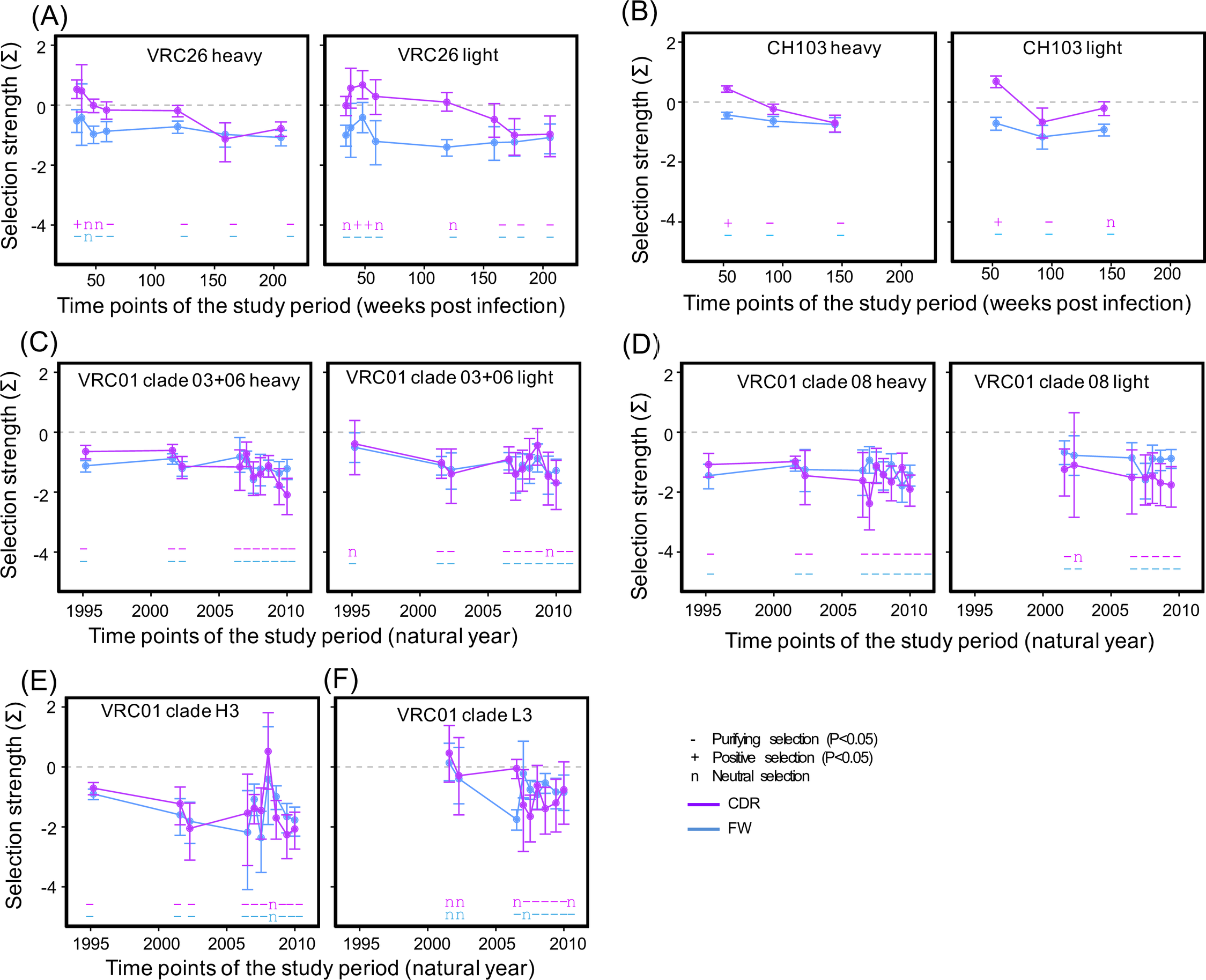

Supplement: S5 Fig — The results are consistent with that of Fig 6. (A) Selection pressure changes of the VRC26 lineage heavy (left) and light (right) chains. (B) Selection pressure changes of the CH103 lineage heavy (left) and light (right) chains. (C) Selection pressure changes of the VRC01 clade 03+06 heavy (left) and light (right) chains. (D) Selection pressure changes of the VRC01 clade 08 heavy (left) and light (right) chains. (E) Selection pressure changes of the VRC01 clade H3. (F) Selection pressure changes of the VRC01 clade L3. (TIF) [file pcbi.1004940.s007.tif]

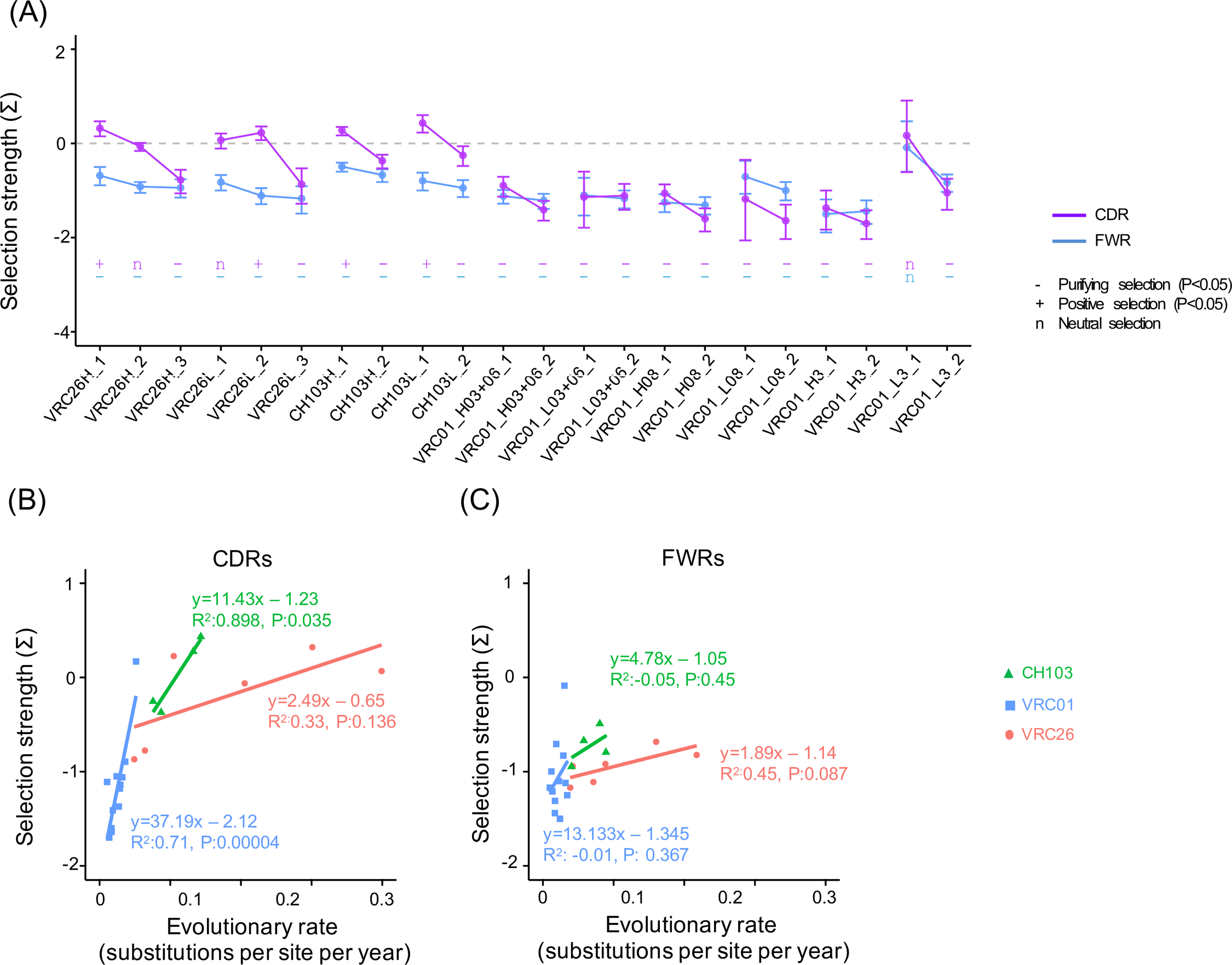

Supplement: S6 Fig — (A) Selection pressure changes over time. (B) Linear correlations between selection pressure and evolutionary rate of CDRs. (C) Linear correlations between selection pressure and evolutionary rate of FWRs. (TIF) [file pcbi.1004940.s008.tif]

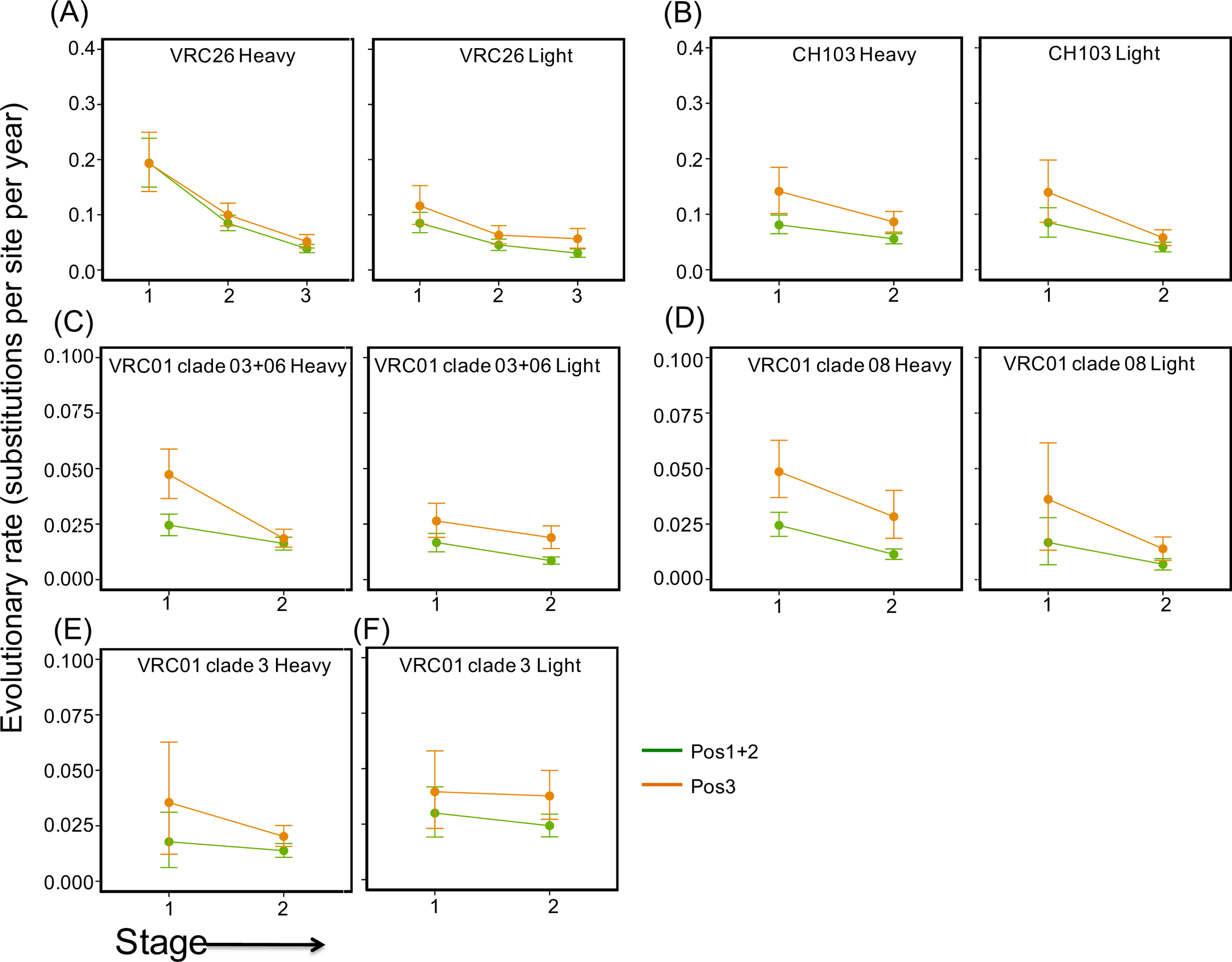

Supplement: S7 Fig — (A) The evolutionary rate changes for the 1st+2nd and the 3rd codon positions of the VRC26 lineage heavy (left) and light (right) chains. (B) The evolutionary rate changes for the 1st+2nd and the 3rd codon positions of the CH103 lineage heavy (left) and light (right) chains. (C) The evolutionary rate changes for the 1st+2nd and the 3rd codon positions of the VRC01 clade 03+06 heavy (left) and light (right) chains. (D) The evolutionary rate changes for the 1st+2nd and the 3rd codon positions of the VRC01 clade 08 heavy (left) and light (right) chains. (E) The evolutionary rate changes for the 1st+2nd and the 3rd codon positions of the VRC01 clade H3. (F) The evolutionary rate changes for the 1st+2nd and the 3rd codon positions of the VRC01 clade L3. The evolutionary rates of both the 1st+2nd and the 3rd codon positions decreased over time, except clade H3, L3, and the 3rd codon positions of two clades (03+06 light chain and both heavy and light chains of clade 08) in the VRC01 lineage. This suggests evolutionary rate is systematically regulated and selection pressure change cannot fully explain the slowing of evolutionary rate. (TIF) [file pcbi.1004940.s009.tif]
